# Structural Diversity, XAS and Magnetism of Copper(II)-Nickel(II) Heterometallic Complexes Based on the [Ni(NCS)_6_]^4−^ Unit

**DOI:** 10.3390/ma16020731

**Published:** 2023-01-11

**Authors:** Natalia Tereba, Tadeusz M. Muzioł, Joanna Wiśniewska, Robert Podgajny, Alina Bieńko, Grzegorz Wrzeszcz

**Affiliations:** 1 Faculty of Chemistry, Nicolaus Copernicus University in Toruń, Gagarina 7, 87-100 Toruń, Poland; 2 Faculty of Chemistry, Jagiellonian University, Gronostajowa 2, 30-387 Kraków, Poland; 3 Faculty of Chemistry, University of Wroclaw, Joliot-Curie 14, 50-383 Wrocław, Poland

**Keywords:** hexaisothiocyanatonickelate(II) complexes, thiocyanato bridges, single crystal XRD, XAS, magnetic properties, spin density

## Abstract

The new heterometallic compounds, [{Cu(pn)_2_}_2_Ni(NCS)_6_]_n_·2nH_2_O (**1**), [{Cu^II^(trien)}_2_Ni(NCS)_6_Cu^I^(NCS)]_n_ (**2**) and [Cu(tren)(NCS)]_4_[Ni(NCS)_6_] (**3**) (pn = 1,2-diaminopropane, trien = triethylenetetramine and tren = tris(2-aminoethylo)amine), were obtained and characterized by X-ray analysis, IR spectra, XAS and magnetic measurements. Compounds **1**, **2** and **3** show the structural diversity of 2D, 1D and 0D compounds, respectively. Depending on the polyamine used, different coordination polyhedron for Cu(II) was found, i.e., distorted octahedral (**1**), square pyramidal (**2**) and trigonal bipyramidal (**3**), whereas coordination polyhedron for nickel(II) was always octahedral. It provides an approach for tailoring magnetic properties by proper selection of auxiliary ligands determining the topology. In **1**, thiocyanate ligands form bridges between the copper and nickel ions, creating 2D layers of **sql** topology with weak ferromagnetic interactions. Compound **2** is a mixed-valence copper coordination polymer and shows the rare ladder topology of 1D chains decorated with [Cu^II^(tren)]^2+^ antennas as the side chains attached to nickel(II). The ladder rails are formed by alternately arranged Ni(II) and Cu(I) ions connected by N2 thiocyanate anions and rungs made by N3 thiocyanate. For the Cu(I) ions, the tetrahedral thiocyanate environment mixed N/S donor atoms was found, confirming significant coordination spheres rearrangement occurring at the copper precursor together with the reduction in some Cu(II) to Cu(I). Such topology enables significant simplification of the magnetic properties modeling by assuming magnetic coupling inside {Ni^II^Cu^II^_2_} trinuclear units separated by diamagnetic [Cu(NCS)(SCN)_3_]^3−^ linkers. Compound **3** shows three discrete mononuclear units connected by N-H…N and N-H…S hydrogen bonds. Analysis of XAS proves that the average ligand character and the covalency of the unoccupied metal d-based orbitals for copper(II) and nickel(II) increase in the following order: **1** → **2** → **3**. In **1** and **2**, a weak ferromagnetic coupling between copper(II) and nickel(II) was found, but in **2**, additional and stronger antiferromagnetic interaction between copper(II) ions prevailed. Compound **3**, as an ionic pair, shows, as expected, a spin-only magnetic moment.

## 1. Introduction

At present, materials chemistry is focused on novel materials with high applicability in the fields such as magnetic and optoelectronic devices, nonlinear optics, luminescence, biological, medicine-related materials or catalysis [1,2,3,4,5,6,7,8,9]. The 3d-electron metal ions are characterized by a large variety of oxidation states, coordination numbers and geometry of coordinating spheres, so their complexes may have interesting redox, magnetic or optical properties [10,11,12]. An important feature of molecular materials such as heterometallic complexes is the ability to create bridges by ligands playing an important role as carriers of magnetic interactions between metal centers. These interactions may significantly affect the magnetic properties of the system compared to identical single metallic units. Molecular materials can be multifunctional compounds, such as magnets, which combine magnetic ordering with chirality, conductivity and porosity, and even with two of such additional properties [1,13,14,15,16,17].

The thiocyanate anion belongs to the widespread bridging ligands, able to connect metal centers using both the nitrogen atom as a hard donor and the sulfur atom as a soft donor [18]. The ambidentate nature, along with the bridging abilities of the thiocyanate ion, gives rise to a number of structures with different dimensions. The use of different paramagnetic metal centers can increase the possibility of the occurrence of interesting magnetic interactions between each other.

Among the 0D compounds, a variety of structures, such as heterometallic thiocyanato bridged binuclear [19], trinuclear [20], pentanuclear [21] complexes and non-bridged ionic pairs [22], deserves attention. The Re^IV^-SCN-M^II^ (M = Mn, Fe, Co, and Ni) bridge transmitting weak antiferromagnetic interactions has been found in [(Me_2_phen)_2_M(μ_1,3_-NCS)Re(NCS)_5_]·CH_3_CN (Me_2_phen = 2,9-dimethyl-1,10-phenanthroline) [19]. The rhenium(IV) ion reveals a distorted [N_5_S] distorted octahedral environment, whereas the M(II) ions are five-coordinated with a distorted trigonal bipyramidal structure. The trinuclear [{M^II^L_N4_}_2_{Mn^II^(NCS)_4_}](ClO_4_)_2_ (M = Cu, Ni; L_N4_ = *N*-*dl*-5,7,7,12,14,14-hexamethyl-1,4,8,11-tetraazacyclotetradeca-4,11-diene) complexes with two equivalent thiocyanato bridges and isostructural trinuclear M^II^-Mn-M^II^ moiety, are an example where a change in M^II^ drastically changes the magnetic properties [20]. The Cu-Mn-Cu compound shows weak ferromagnetic interactions, whereas the Ni-Mn-Ni compound contains diamagnetic five-coordinated, square pyramid nickel(II) and, from a magnetic point of view, behaves as mononuclear manganese(II) complexes. Two isostructural pentanuclear complexes [{ML_N4_}_3_{Mn(NCS)_5_}_2_] (M = Ni, Cu) are constructed from two trigonal bipyramidal [Mn(NCS)_5_]^3–^ double bridging entities, one central octahedral M(II) moiety and two terminal M(II) five-coordinated square pyramids [21]. Both complexes show weak antiferromagnetic interactions. The ion-pair [Cu(tren)(NCS)]_4_[Mn(NCS)_6_] complex has been reported with negligible magnetic coupling between paramagnetic ions [22].

A number of 1D thiocyanato bridged heterometallic complexes are known, among them [{M(en)_2_}{Pt(SCN)_4_}]_n_ (M = Ni, Cu) [23]. *Cis* and *trans* fashion at the [Pt(SCN)_4_]^2−^ bridging unit occurs in two forms of the copper(II)–platinum(II) compound. On the other hand, nickel(II)–platinum(II) compound occurs only in the *cis* form. The one-dimensional [Cu(en)(µ_1,3_-NCS)_4_Hg]_n_ compound consists of ladder-like zigzag ribbons with three different, {Cu(en)(µ_1,3_-NCS)_2_Hg}, ({Cu(en)(µ_1,3_-NCS)_2_Hg})_2_ and ({Cu(en)(µ_1,3_-NCS)_3_Hg})_2_, macrocycles [24]. Three different types of 1D chain crystal structures in the [Cu(diamine)_2_Zn(NCS)_4_]_n_·n(solv) (diamine = en, pn) systems have been described, namely zigzag, helical or quasi-linear [25,26,27]. Additionally, two modes of thiocyanate bridges, µ_1,3_-NCS (more common) and µ_1,1_-SCN, have been found among these compounds. A series of 1D ferrimagnetic systems have been reported for coordinated polymers based on [Mo(NCS)_6_]^3−^ in combination with nickel(II) and cobalt(II) complexes [28].

A few examples of heterometallic 2D structures bridging by thiocyanate ligands with ferromagnetic coupling between metal centers are known [29,30,31]. The first molecular magnet based on thiocyanate bridging heterometallic 2D complex has been reported [32].

Among a few known 3D thiocyanato-bridged heterometallic complexes, [NH_4_]_2_[NiCd(SCN)_6_] with double perovskite-type structure was reported, which shows reversible ordered-disordered phase transition [33]. Recently, M.J. Cliffe et al. [34] described, inter alia, the structure of ordered defects in the NH_4_^+^-Ni(II)-NCS^−^-Bi(III) system, which appears as Prussian Blue analog.

There are only about twenty literature reports on compounds with heksathiocyanato-*N*-nickelate(II) unit [33,34,35,36,37,38,39,40,41,42,43,44,45,46,47,48,49,50,51,52], even fewer with known crystallographic structure [33,34,35,36,37,38,39,40,41,42,43,44,45,46,47], mostly with an organic cation as a counterion [37,38,39,40,41,42,43,44,45,46,47]. Such hybrid systems are interesting due to their particular properties. For example, they show ferro- and piezoelectric properties [39] or four-step thermosensitive dielectric response directly connected with reversible structural phase transitions [47]. Such compounds can be ionic liquids with thermochromic properties [44,51], frequency-regulated dielectric switches [42] or semiconductors [43]. On the other hand, reports on heterometallic complexes based on [Ni(NCS)_6_]^4−^ are extremely rare [33,34,40,48,53]. J.-H. Bi et al. [40] described the single-crystal X-ray structure of [Co^II^(phen)_3_]_2_[Ni(NCS)_6_]. Two subsequent reports [48,49] do not contain the SC-XRD structure. A. Tomkiewicz et al. [48] studied the magnetic properties of copper(II)–nickel(II) heksathiocyanato-*N*-nickelate(II) based compound. Whereas A.R.S. Rad et al. [49] prepared zinc(II)–nickel(II) precursor for water-gas shift reaction catalyst. There are also a few complexes with the formula [Ni(NCS)_6-n_Ni(SCN)_n_]^4−^ e.g., with mixed N-and S-donor thiocyanate [53,54,55,56], but this is not the focus of this work. SC-XRD structure and properties of the penta-nuclear homometallic complex based on [Ni(NCS)_6_]^4−^ were reported [35].

With the above in mind, we extended our previous research interest to heterometallic compounds containing anionic blocks with *N*-coordinated thiocyanate ions, e.g., [Cr(NCS)_6_]^3−^ [57,58,59,60,61,62,63,64], [Cr(NCS)_4_(NH_3_)_2_]^−^ [65] and [Zn(NCS)_4_]^2−^ [26,27], and involve rarely used [Ni(NCS)_6_]^4−^ building block. We present synthesis, SC-XRD crystal structure, X-ray absorption spectra and magnetic properties of three new [Ni(NCS)_6_]^4−^ based compounds, with different dimensionality and variety of Cu oxidation states and polyhedral coordination geometries: [{Cu(pn)_2_}_2_Ni(NCS)_6_]_n_·2nH_2_O (**1**), [{Cu^II^(trien)}_2_Ni(NCS)_6_Cu^I^(NCS)]_n_ (**2**) and [Cu(tren)(NCS)]_4_[Ni(NCS)_6_] (**3**). It should be noted that relatively rarely used XAS can provide valuable information concerning electron structure and local geometry, coordination spheres content and coordination number, as well as bond lengths [66]. Those spectra are sensitive to oxidation number, electron configuration and differential orbital covalency.

## 2. Materials and Methods

Materials: All reagents used in the synthesis were of analytical grade and used without further purification. All polyamine ligands were purchased from Sigma Aldrich (Darmstadt, Germany) and other reagents from Avantor Performance Materials Poland SA (formerly POCH, Gliwice, Poland) and Chempur (Piekary Śląskie, Poland).

### 2.1. Synthesis of [{Cu(pn)_2_}_2_Ni(NCS)_6_]_n_·2nH_2_O (1)

An amount of 241 mg of Cu(NO_3_)_2_∙3H_2_O (1 mmol) was dissolved in 10 mL of water. Then, 0.26 mL of pn (3 mmol) was slowly added with constant stirring. The violet solution was obtained. In other beakers, 295 mg of Ni(NO_3_)_2_∙6H_2_O (1 mmol) and 324 mg of NaNCS (4 mmol) were dissolved in 2 and 1 mL of water, respectively. They were mixed and added to the copper–amine solution. The mixture was stirred at room temperature for 15 min. There was no precipitate, and the clear solution was left to evaporate. The violet crystals, suitable for X-ray analysis, were obtained after a few days. The analysis found the following: C, 25.20; H, 5.37; N, 22.79%. Calc. for C_18_H_44_N_14_O_2_S_6_NiCu_2_: C, 24.94; H, 5.12; N, 22.62%. Selected IR bands (cm^−1^): ν(OH) 3578 m, ν(NH) 3451 m, 3299 m, 3224 m, ν(CH) 2972 w, 2879 w, ν(CN) 2106 vs,br, δ(NH_2_) 1582 s, δ(CH_2_) 1457 m, 1380 m, ν(CN) 1064 s, 1019 s, ν(CC) 932 m, 772 w, ν(CS) 835 w, δ_rocking_(NH_2_) 688 m, δ(NCS) 487 m.

### 2.2. Synthesis of [{Cu^II^(trien)}_2_Ni(NCS)_6_Cu^I^(NCS)]_n_ (2)

A total of 341 mg of CuCl_2_·2H_2_O (2 mmol) was dissolved in 10 mL of water. A solution of 0.30 mL trien (2 mmol) in 20 mL of water was prepared separately. Both solutions were mixed, and a violet solution was formed. An amount of 238 mg of NiCl_2_·6H_2_O (1 mmol) in 10 mL of water H_2_O and 583 mg of KNCS (6 mmol) in 6 mL of water were mixed. Then, the nickel(II) solution was added to the copper(II) solution. A violet-navy solution was formed. The navy-blue crystals, suitable for X-ray analysis, were obtained after about a month. The analysis found the following: C, 24.94; H, 4.52; N, 22.57%. Calc. for C_19_H_36_N_15_S_7_NiCu_3_: C, 24.06; H, 3.83; N, 22.15%. Selected IR bands (cm^−1^): ν(NH) 3441 m, 3306 m, 3268 m, 3171 s, ν(CH) 2962 w, 2940 w, 2875 w, ν(CN) 2116 s, 2071 s, δ(NH_2_) 1581 s, δ(CH_2_) 1451 m, ν(CN) 1107 m, 1089 m, 1055 m, 1018 s, ν(CC) 970 m, ν(CS) 869 m, δ_rocking_(NH_2_) 624 m, 607 m, δ(NCS) 478 m.

### 2.3. Synthesis of [Cu(tren)(NCS)]_4_[Ni(NCS)_6_] (3)

To 341 mg (2 mmol) of CuCl_2_∙2H_2_O in 10 mL of distilled water, 20 mL of tren (0.30 mL, 2 mmol) was added. The solution turned from light blue to blue. An amount of 238 mg (1 mmol) of NiCl_2_∙6H_2_O was dissolved in 10 mL of water, and 583 mg (6 mmol) of NaNCS in 6 mL of water was added. They were mixed and added to the copper–amine solution. The mixture was stirred at room temperature for 30 min, and then the solution was filtered. The green-blue crystals, suitable for X-ray analysis after one month, were obtained. The analysis found the following: C, 27.64; H, 5.43; N, 24.78%. Calc. for C_34_H_72_N_26_S_10_NiCu_4_: C, 27.62; H, 4.91; N, 24.63%. Selected IR bands (cm^−1^): ν(NH) 3351 m, 3326 m, 3301 m, 3235 s, 3129 m, ν(CH) 2955 w, 2872 w, ν(CN) 2100 vs,br, δ(NH_2_) 1587 s, δ(CH_2_) 1471 m, 1362 m, ν(CN) 1057 s, 1031 m, 984 s, ν(CC) 901 m, 775 m, ν(CS) 867 w, δ_rocking_(NH_2_) 618 m, δ(NCS) 473 m.

### 2.4. Methods

Elemental analysis was performed on Vario Macro CHN Analyzer (Elementar Analysensysteme GmbH, Langenselbold, Germany). Infrared spectra were recorded on a Vertex 70v Spectrometer from Bruker Optik: GmbH (Ettlingen, Germany) in the range 4000–400 cm^−1^. Magnetic measurements in the temperature range of 1.8–300 K were performed using SQUID MPMS-3 and MPMS-XL-5 magnetometers at the magnetic field of 0.1 and 0.5 T for **1** and **2**, respectively. The data were corrected for the sample holder and the underlying diamagnetism using Pascal’s constants [67] (−480 and −485 × 10^−6^ cm^3^ mol^−1^, for compounds **1** and **2**, respectively). The temperature-independent paramagnetism and all uncertainty in diamagnetic correction were treated as variable parameters within the fitting procedures (see below). The effective magnetic moment was calculated from the equation: *µ_eff_* = 2.828 (*χ_M_^corr^ ∙ T*)^1/2^. Magnetization versus magnetic field measurements were carried out at 1.8 K for **1** and 2 K for **2** in the magnetic field range 0–7 Tesla. Magnetic susceptibility for **3** was measured at room temperature by the Faraday method on a homemade balance at field strength up to 1.0 T. The magnetic field was calibrated with Hg[Co(NCS)_4_] [68]. The X-ray absorption spectra were recorded at the National Synchrotron Radiation Centre SOLARIS at the bending magnet PEEM/XAS beamline for N (350–450 eV) and O (500–550 eV) K-edge as well as Ni (850–900 eV) and Cu (910–1000 eV) L-edge. Samples of **1**, **2** and **3** were finely ground and attached to double-sided adhesive conductive graphite tape. For all measurements, the step size of 0.2 eV was used for the edge regions and 0.4 eV for the remaining regions. The data sets were collected at room temperature under ultra-high vacuum (UHV) in the total electron yield mode (TEY). The data were processed using the PyMca 5.4.0 program package and were evaluated using the OriginPro 5.0 (Northampton, MA, USA) program as the data analysis and graphing software.

### 2.5. Single Crystal X-ray Diffraction Measurement

The diffraction experiments for the single crystal of **1**, **2** and **3** were performed at room temperature using an Oxford Sapphire CCD diffractometer, MoKα radiation λ = 0.71073 Å. During the data processing, the numerical absorption correction was applied [69]. The structure was solved by the direct methods and refined with a full-matrix least-squares procedure on F^2^ (SHELX-97 [70]). For all heavy atoms, a refinement procedure with anisotropic displacement parameters was applied. Positions of hydrogen atoms attached to carbon atoms were determined from geometrical conditions and refined with thermal displacement parameters fixed to a value of 20% or 50% higher than those of the corresponding carbon atoms. Hydrogen atoms from NH and NH_2_ groups, as well as water molecules, were located in different electron density syntheses. In (**1**), in the refinement process, hydrogen atoms from the O7 water molecule were refined with constraints (DFIX and DANG) to ensure their reasonable geometry. In (**2**), ISOR restraints were applied to occupy the S7 atom partially. All figures were prepared in DIAMOND [71] and ORTEP-3 [72]. The results of the data collection and refinement are summarized in Table 1. CCDC 2164900, 2164901 and 2164903 contain the supplementary crystallographic data for **1**–**3**, respectively. These data can be obtained free of charge from The Cambridge Crystallographic Data Centre via www.ccdc.cam.ac.uk.data_request/cif.

## 3. Results and Discussion

### 3.1. Infrared Spectra

The IR spectra are a good and convenient criterion to determine the coordination modes of thiocyanate ligands and to prove the presence of polyamine ligands and the lattice water [73,74,75]. In the infrared spectra of **1**, **2** and **3,** very intense bands at *ca*. 2100 cm^−1^ were detected, coming from CN stretching vibration of bridging thiocyanate. For **1** and **3**, the band is very broad and does not show splitting, whereas for **2,** two peaks at 2116 cm^−1^ and at 2071 cm^−1^ were distinguished, assignable to the NCS^−^ bridge and to the terminal N-bonded thiocyanate, respectively. The NCS^−^ bending vibration appears in all studied spectra as bands in the range 473–478 cm^−1^. The bands occurring in the range 835–869 cm^−1^ are due to the vibration of the CS group, also coming from the thiocyanate ligand. Some of the expected low-intensity new CS bands can be masked by stronger bands from the rocking NH_2_ vibrations. Several bands occurring in the range 3451–3129 and 2972–2872 cm^−1^ are characteristic of the polyamine group, NH and CH vibrations, respectively. The peak occurring in the IR spectrum of **1** at 3578 cm^−1^ is due to the stretching vibration of the OH group from water molecules.

### 3.2. Structure of [{Cu(pn)_2_}_2_Ni(NCS)_6_]_n_·2nH_2_O (1)

**Figure 1:**
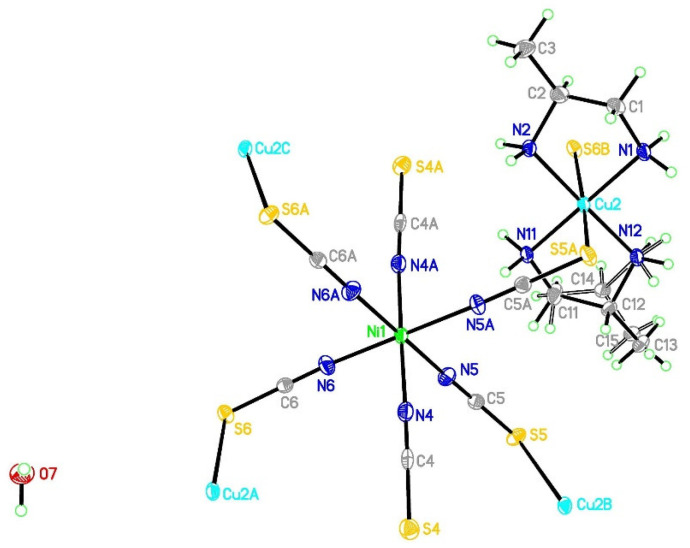
The local molecular structure of [{Cu(pn)_2_}_2_Ni(NCS)_6_]_n_∙2nH_2_O (**1**) drawn at the 30% probability level. N4A, N5A and N6A denote symmetrically related thiocyanate anions.

The reported complex crystallizes in a monoclinic C2/c space group with the nickel ions positioned at a twofold axis. In the asymmetric unit, there are copper and nickel ions, three linear thiocyanate ligands (N-C-S angles: 178.9(4)–179.6(4)°), two 1,2-diaminepropane molecules and one water molecule (Figure 1). The selected bond lengths and angles are presented in Table 2 and Table S1, Supplementary Materials. Carbon atoms in pn molecules reveal positional disorder with occupancy 0.55 (C12 and C13) and 0.45 (C14 and C15). Copper(II) ions adopt 4+2 coordination with four short Cu-N and two much longer Cu-S bonds. The Cu-N bonds are found in the range 2.003(3)–2.028(3) Å and are similar to the values found in other complexes [27,31,76,77,78]. The axial bonds are distinctly longer than equatorial bonds and are equal to 2.9883(11) and 3.0224(10) Å for Cu-S5 and Cu-S6, respectively. Therefore Cu-N and Cu-S distances are characteristic of the geometry of the elongated octahedron [31,79]. Nickel(II) ion was found in a slightly distorted octahedral environment, with six Ni-N bonds being as follows: 2.060(3), 2.073(3) and 2.118(4) Å. These values correspond to those found in the literature [44,54]. The longest distance was found for the non-bridging thiocyanate ions indicating that a bridge formation resulted in a shortening of Ni-N bonds. The selected valence angle description is given in the Supplementary Materials.

**Figure 2:**
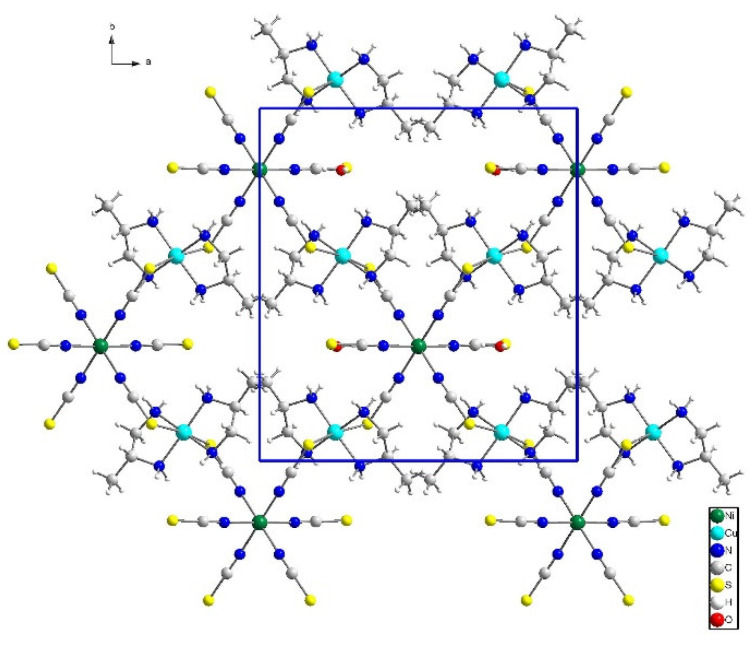
Structure of single *ab* layer along *c* axis in **1**. For clarity of the figure, minor population sets are omitted.

In crystal packing, there are *ab* layers of sql topology [80] composed of covalently connected copper(II) and nickel(II) blocks, with four thiocyanate ions involved in bridge formation between paramagnetic centers (Figure 2 and Figure S1, Supplementary Materials). In such a layer, every copper(II) ion is connected with two nickel(II) ions, and each nickel(II) cation serves as a node of the network interacting with four copper(II) cations. The *ab* layers form …ABAB… stack motifs with adjacent layers shifted by *ca*. [0, 0.45y, −0.5z], and hence, the nodes of A layer are not positioned above the cavity center of the B layer (Figure S2, Supplementary Materials). It is noted that the layer topology is identical to that in the 2D compound of [{Cu(pn)_2_}_2_Mn(NCS)_6_]_n_∙2nH_2_O [31].

The shortest heterometallic distances are 5.616 and 5.708 Å between Ni(II) and Cu(II) cations connected by four bridging thiocyanate ions located at equatorial positions, whereas the apical positions are occupied by the non-bridging thiocyanate forming the longest Ni-N bonds. These distances for Ni and Cu ions connected via thiocyanate bridge are shorter than in [CuL_N4_{Ni(NCS)_4_(H_2_O)_2_}]_n_ (6.342 Å) [81] and [Cu(oxpn)Ni(μ-NCS)(H_2_O)(tmen)]_2_(X)_2_ (oxpn = *N*,*N’*-bis(3-aminopropyloxamide)) (6.313 and 6.335 Å for X = PF_6_^−^ and X = ClO_4_^−^, respectively) [82]. In **1**, the Cu-Cu distance between copper(II) ions from adjacent layers are similar to those inside the layer (e.g., are 7.284, 7.987 Å) and is 7.638 Å. The Ni-Ni distance between nickel(II) ions in one layer is 11.317 Å, while the distance between nickel(II) ions from the adjacent layers is significantly smaller (9.214 Å).

Most of the observed hydrogen bonds (N-H…N/S, O-H…N/S, N-H…O) were found inside the *ab* layer between N1, N2 and N12 NH_2_ groups and N4 and S4 atoms of non-bridging thiocyanate anion as well as the O7 water molecule located in a cavity of *ab* layer (Table S2, Supplementary Materials). Moreover, these interactions may account for the slight elongation of Ni1-N4 according to Ni1-N5 and Ni1-N6 bonds. There were found only two interlayer hydrogen bonds: O7-H7A…S6[x, 1−y, −1/2+z] and N11-H11B…S5[−x, −y, 1−z]. In the former case, interactions are mediated by the O7 water molecule, whereas in the latter, direct contact between paramagnetic lattices exists. Thus, it can be assumed that the layers interact with each other affecting the observed antiferromagnetic coupling.

### 3.3. Structure of [{Cu^II^(trien)}_2_Ni(NCS)_6_Cu^I^(NCS)]_n_ (2)

**Figure 3:**
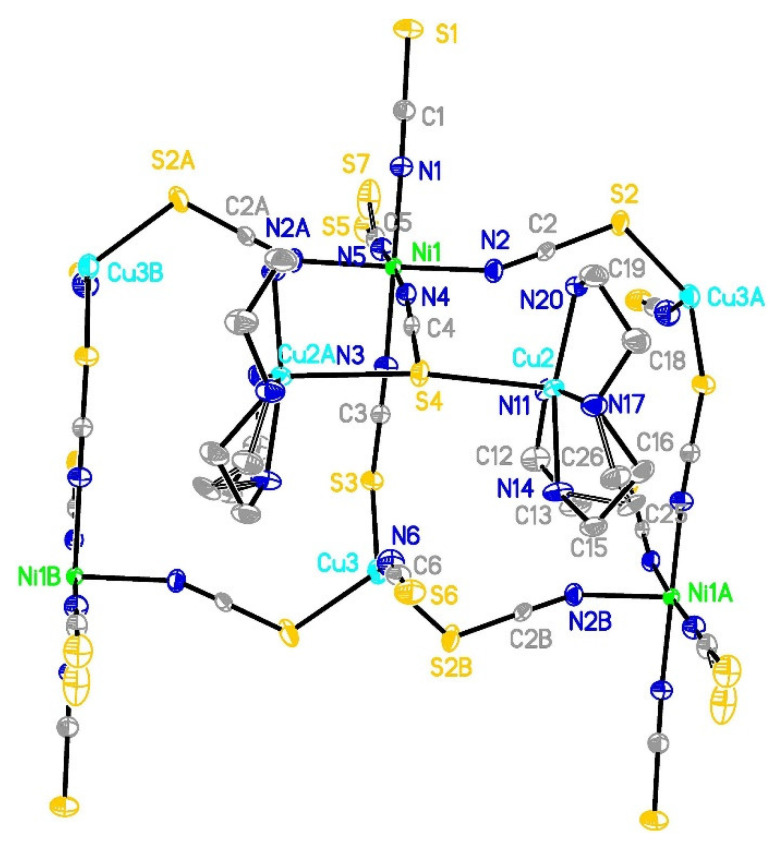
The structure of [{Cu^II^(trien)}_2_Ni(NCS)_6_Cu^I^(NCS)]_n_ (**2**) with thermal ellipsoids at the level of 30% probability. Hydrogen atoms are omitted for clarity.

Compound **2** crystallizes in the orthorhombic Pnma space group with Ni1 and Cu3 metal ions as well as all thiocyanates except NCS(2) anion positioned at the m plane. Hence, in the asymmetric part of the unit cell, there are two copper ions (sof = 0.5 for Cu3 and 1.0 for Cu2), nickel(II) ion (sof = 0.5), one trien molecule and 3.5 thiocyanate anions (Figure 3). The selected bond lengths and angles are presented in Table 3 and Table S3, Supplementary Materials. The Cu:Ni ratio of 3:1 was also confirmed by SEM-EDX analysis. For S5, C15 and C16 atoms, the positional disorder was detected, and alternate positions were refined with occupancies of 0.4:0.1, 0.65:0.35 and 0.65:0.35, respectively. Ni1 is found in a slightly distorted octahedral environment composed of six nitrogen atoms coming from thiocyanate anions with Ni-N distances ranging from 2.048(3) to 2.103(3) Å (Table 3). Two copper positions differ in coordination number. Cu3 atom is found in a slightly distorted tetrahedral environment (τ_4_ = 0.89, τ_4_‘ = 0.87) [83,84] with three sulfur atoms from thiocyanate anions with Cu-S bonds from 2.3520(8) to 2.3711(14) Å and N6 atom (1.946(5) Å) forming the coordination sphere. Cu2 coordination environment can be described as a square pyramid (τ_5_ = 0.080) [85] with four nitrogen atoms from a trien molecule (Cu-N: 1.999(2)–2.029(4) Å) in the base and apical position occupied by S4 atom (2.6422(4) Å). Cu2 is shifted by 0.275 Å from the basal plane. In this compound, outer NH_2_ groups form slightly longer Cu(II)-N bonds than the inner NH groups. These bond lengths are similar to [Cu(trien)(SCN)](NCS) (Cu-N: 2.007–2.030 Å, Cu-S: 2.606 Å) [86]. The broader range of Cu-N_trien_ bond lengths was observed in [Cu(trien)(NCS)]^+^ moieties (1.975–2.094 and 1.982–2.064 Å) [87]. In [Cu(AA)(trien)](ClO_4_)_2_ (AA = bpy, phen) moieties, high conformational flexibility of trien resulted in octahedral coordination with significant elongation of one outer Cu-N_trien_ (2.670 and 2.723 Å for phen and bpy, respectively) bond due to the Jahn–Teller effect [88]. Similar Cu-N_trien_ bonds (1.996–2.019 Å) were noted for a homonuclear coordination polymer with azide bridges [89]. The selected valence angle description is given in the Supplementary Materials.

**Figure 4:**
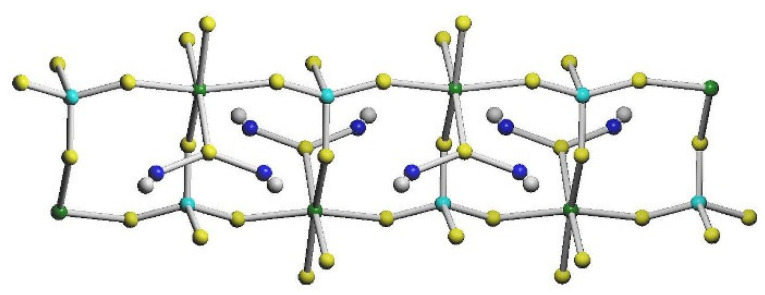
The ladder topology in **2** with Cu(II) in blue, Cu(I) in cyan, nickel in green, thiocyanates in yellow and trien ligands in grey.

Two important points should be mentioned. First, analysis of coordination spheres and atomic charges indicated that Cu(II) occupies a pentacoordinate position, whereas Cu(I) is located in a tetrahedral environment. This indicates that a redox process occurs during the reaction and/or crystallization, associated with the oxidation of some thiocyanates and reduction in Cu(II). As a result, a stable [Cu^I^(NCS)(SCN)_3_]^3−^ unit is formed, which is coordinated terminally (N2 and N3) to Ni(1) atom. Second, the ladder running along the *b* axis is formed due to three thiocyanates bridging Ni(II) and Cu(I) cations with two symmetrically related S2 thiocyanate anions involved in ladder rails and S3 forming rungs between Ni1 and Cu3 atoms (Figure 4). Hence, [Cu^II^(trien)]^2+^ moieties are located in pending side chains connected to Ni(II) via S4 thiocyanate with an S4 atom forming also a direct bridge between two Cu(II) units (Figure 3).

Despite complicated ladder topology, the magnetic description might exploit a relatively simple model of exchange interactions as [{Cu^II^(trien)}_2_{Ni^II^(NCS)_3_}]^3+^ units are separated by diamagnetic [Cu^I^(NCS)(SCN)_3_]^3−^ linkers (see Section 3.6). We can presume that the {Ni^II^Cu^II^_2_} trinuclear unit bridged by S4 thiocyanate anion should be sufficient. Three metals involved in interaction form T shape motif (with NCS(4) anion acting as a linker) with 90.08(2)° for C4-S4-Cu2 and 171.19(5)° for Cu2[x, −y+1/2, z]-S4-Cu2.

In this structure, S1 and S5 thiocyanates are coordinated terminally to Ni(II), whereas S6 to Cu(I) and S2, S3 and S4 anions are involved in bridge formation. Thiocyanate coordination geometry might be described by Cu-S-C and Cu/Ni-N-C angles. Cu^II^-S-C angles range from 108.30(9) to 109.57(16)°, whereas for T-shaped S4 linker this value is much smaller (90.08(2)°). Cu/Ni-N-C angles range from 154.1(2) to 178.7(4)° with larger values for the N-terminal ligand, while the smallest angle was observed for the N,S-bridging S2 thiocyanate involved in ladder propagation.

The shortest intermetallic distances important for magnetic properties are found inside the ladder, with Cu2-Cu2[x, 1/2−y, z] and Ni1-Cu2[x, y, z] distances being 5.269 and 5.504 Å, respectively. The parallel running ladders form a tightly packed system (67.4% [90]) connected by N17-H17A…S1[−1/2+x, 1/2−y, 3/2−z], N20-H20A…S5[3/2−x, −y, 1/2+z] and N20-H20A…S7[3/2−x, −y, 1/2+z] hydrogen bonds (Table S4, Supplementary Materials).

### 3.4. Structure of [Cu(tren)(NCS)]_4_[Ni(NCS)_6_] (3)

**Figure 5:**
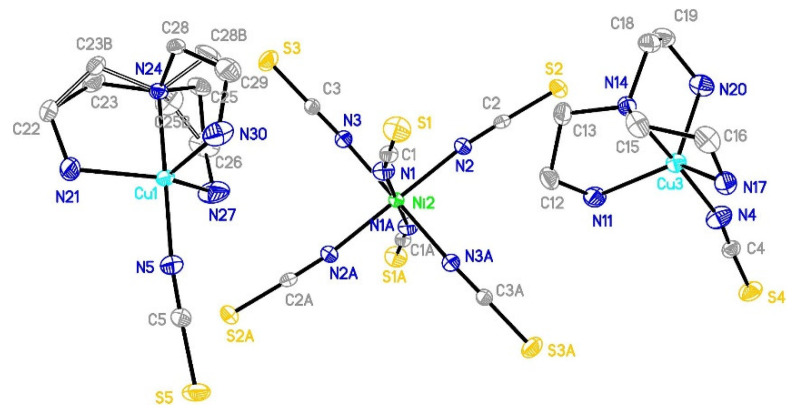
The structure of [Cu(tren)(NCS)]_4_[Ni(NCS)_6_] (**3**) with the thermal ellipsoids at 30% probability. Hydrogen atoms are omitted for clarity.

Compound **3** crystallizes in a monoclinic P 2_1_/c space group with Ni2 ion located at the inversion center, and the Cu:Ni ratio is 4:1 (Figure 5). The selected bond lengths and angles are presented in Table 4 and Table S5, Supplementary Materials. The positional disorder (55:45) is observed for carbon atoms of the tren (N24) molecule. Ni1 is found in a slightly distorted octahedral environment formed by six nitrogen atoms of thiocyanate anion. Ni-N distances range from 2.075(3) to 2.102(3) Å. There are two [Cu(tren)(NCS)]^+^ units with pentacoordinate copper(II) cations with τ_5_ values (0.773 and 0.877 for Cu1 and Cu3, respectively) [85], indicating the distorted trigonal bipyramidal environment with threefold axes of both Cu1 and Cu3 coordination spheres inclined by 58.0°. Both Cu(II) coordination spheres consist of five nitrogen atoms—four coming from the tripodal ligand and one of the thiocyanate anion. Three nitrogen atoms from pendant -CH_2_-CH_2_-NH_2_ groups are found in the equatorial plane with Cu-N_tren_ distances from 2.041(3) to 2.128(3) and from 2.061(3) to 2.109(3) Å for Cu1 and Cu3 coordination spheres, respectively. The apical positions are occupied by central N14 (2.042(2) Å) or N24 (2.034(2) Å) atoms from tren molecules and N4 (1.950(3) Å) or N5 (1.941(3) Å) atoms from thiocyanates forming the shortest bonds in Cu(II) coordination spheres. Very similar coordination spheres were found in isomorphic [Cu(tren)(NCS)]_4_[Mn(NCS)_6_] complex (1.941(2)–2.156(2) Å) [22], mononuclear [Cu(NO_2_)(tren)]_2_(PF_6_)_2_ complex (2.0410(19)–2.1352(17) Å) [91] and [Cu(tren)(acv)]X_2_ (acv = acyclovir, X = NO_3_^−^, ClO_4_^−^, 2.050(4)–2.149(5) and 2.0538(19)–2.1256(19) Å, respectively) (2.149(5) and 2.1256(19) Å) [92]. In [Mo^IV^(CN)_2_(CN-Cu(tren))_6_]^8+^, where the copper units are radially connected to the central molybdenum(IV) cations, Cu-N_tren_ bonds in the copper coordination spheres show a much bigger range with one very short contact (usually below 2.0 Å) and other spanning from 2.03 to 2.15 Å [93]. For trinuclear unit coupled into hexanuclear [Cu_6_(tren)_2_(N_3_)_12_] clusters arranged in centrosymmetric zigzag instead of the long bond, there is one very short bond between lateral -CH_2_-CH_2_-NH_2_ chain and copper(II) ion (1.997(6) to 2.065(5) Å) [94]. The selected valence angle description is given in the Supplementary Materials.

**Figure 6:**
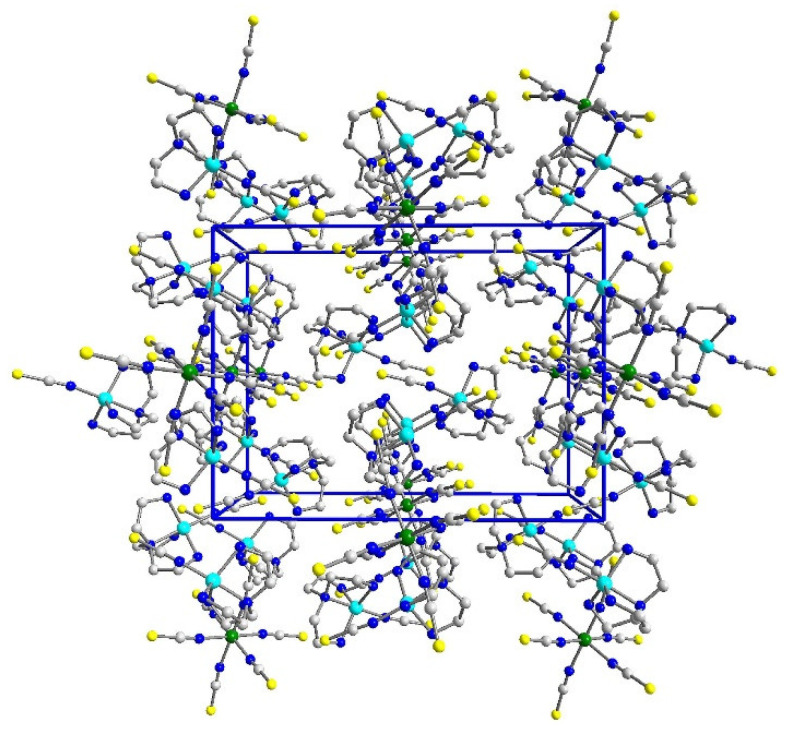
Packing of **3** along the *a* axis shows columns of the copper and nickel units.

The shortest intermetallic distances are created between Cu1 and Cu3[−x, 1−y,−z] ions (5.653 Å), whereas for Cu-Ni, it is 6.913 Å. In the crystal network, the copper and nickel units are tightly packed (packing index: 67.1% [90]), forming columns running along the *b* axis, which are connected by three hydrogen bonds N17-H17A…S1[−x,1−y,−z], N17-H17B…N5[−x,1−y,−z] and N27-H27B…S2[1−x,1−y,−z] (Figure 6, Table S6, Supplementary Materials). The differences in fingerprints for both Cu(II) units prove that they form slightly different interactions in the network (Figure S3, Supplementary Materials). However, in both cases, the most numerous are H…H, S…H and N…H interaction (data in brackets) [95,96].

### 3.5. X-ray Absorption Spectroscopy

**Figure 7:**
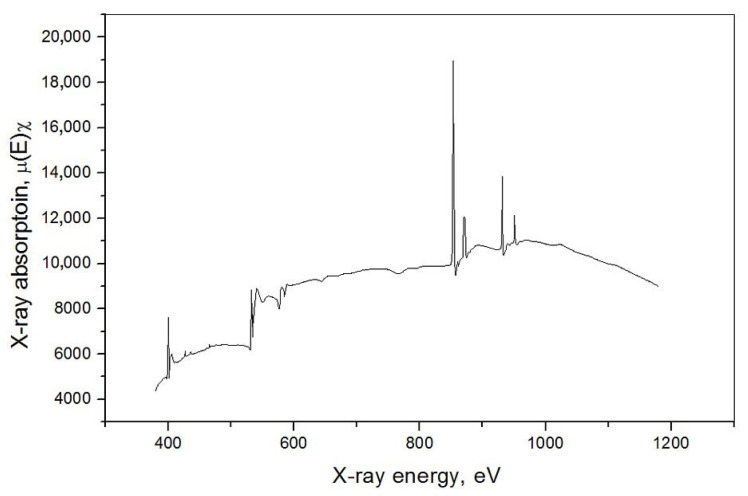
The XAS spectra of [{Cu(pn)_2_}_2_Ni(NCS)_6_]_n_∙2nH_2_O (**1**).

**Figure 8:**
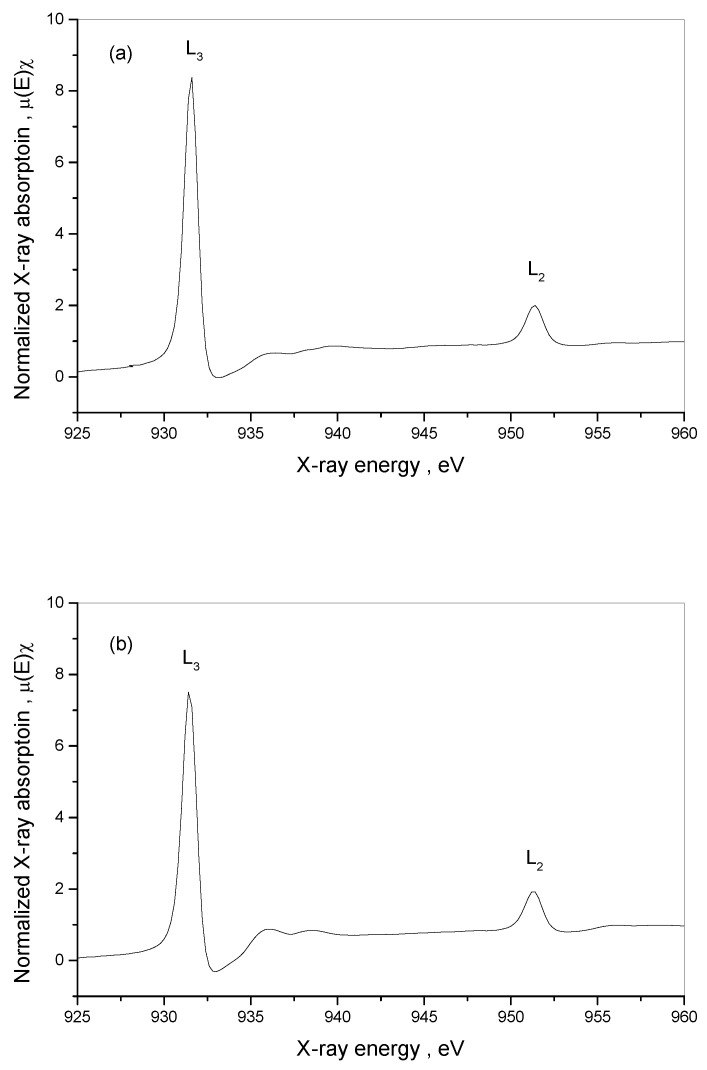

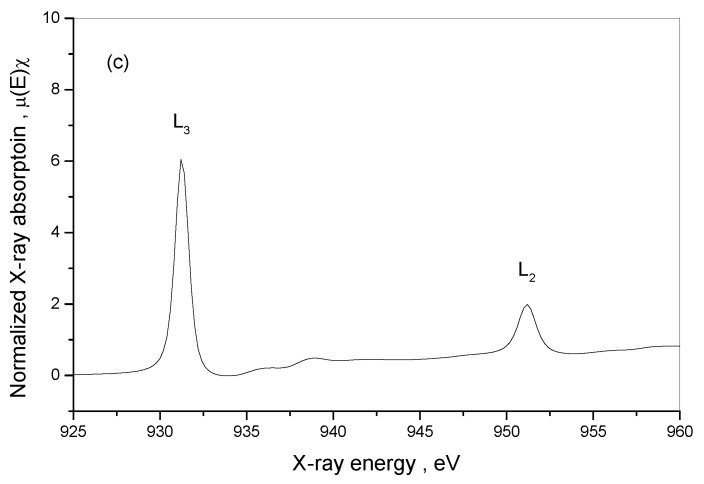
Normalized Cu L-edge absorption spectra for [{Cu(pn)_2_}_2_Ni(NCS)_6_]_n_∙2nH_2_O (**1**) (**a**), [{Cu^II^(trien)}_2_Ni(NCS)_6_Cu^I^(NCS)]_n_ (**2**) (**b**) and [Cu(tren)(NCS)]_4_[Ni(NCS)_6_] (**3**) (**c**).

The XAS measurements showed the selected 3d metals (Ni, Cu) L-edge and the N and O K-edge structure for **1**, **2** and **3** compounds (Figure 7, Figures S4–S6, Supplementary Materials). The L-edge XAS spectra are supposed to allow for determining the differential orbital covalency and the electron structure of metal ion bonds in complexes, which allow for characterizing the metal–ligand interactions and may improve the interpretation of magnetic properties of newly synthesized materials. The normalized Cu L-edge X-ray absorption spectra of **1**, **2** and **3** are presented in Figure 8. The Cu L-edge spectrum is related to the Cu 2p_3/2_→3d (L_3_) and 2p_1/2_→3d (L_2_) transitions and consists of two peaks split by ~20 eV with an intensity ratio above 2: 1 [97]. Three features of L-edge XAS spectra, namely total intensity, energy shift and spectral shape, represent the bonding properties. Within the series **1**–**3**, the integrated intensity of the L_3_-edge transition peak decreases in the order **1** → **2** → **3** indicating that the average ligand character, and therefore the covalency of the unoccupied metal d-based orbitals, increases in the order **1** → **2** → **3** (Table 5). The trend results from changes in the symmetry of copper(II) surrounded by nitrogen atoms of various amines and, in some cases, also by sulfur atoms of bridging thiocyanates. Metal L-edge energy pattern is dependent on three factors: the charge on the absorbing metal atom in the molecule (*Z*_eff_), the ligand field splitting of the d-orbitals, and any difference in the nature of the ligand valence orbitals. In the case of Cu(II), similarly to the high-spin Fe(III) [98], the contribution of the ligand field is weak and should be negligible; the energy shift for the 2p_3/2_→3d transitions decreases in the same order as the integrated intensity of L_3_-edge: **1** → **2** → **3**, and the maximum energy shift is only 0.4 eV in the L-edge spectra between complexes **1** and **3** (Table 5). This is in contrast to low-spin Fe(III) systems with t_2g_-e_g_ energy splitting reaching even 1.4 eV [99].

**Figure 9:**
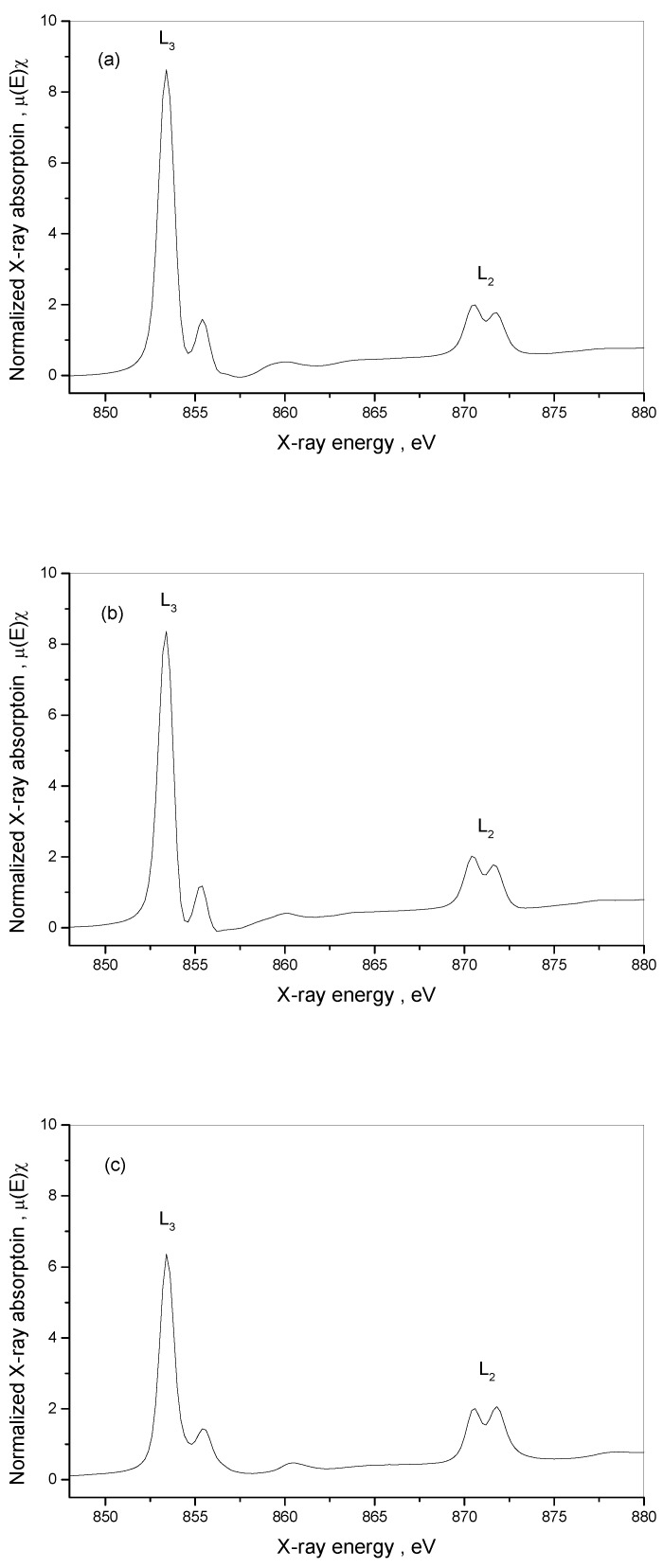
Normalized Ni L-edge absorption spectra for [{Cu(pn)_2_}_2_Ni(NCS)_6_]_n_∙2nH_2_O (**1**) (**a**), [{Cu^II^(trien)}_2_Ni(NCS)_6_Cu^I^(NCS)]_n_ (**2**) (**b**) and [Cu(tren)(NCS)]_4_[Ni(NCS)_6_] (**3**) (**c**).

The normalized Ni L-edge X-ray absorption spectra of **1**, **2** and **3** are presented in Figure 9. The spectra are split into an L_3_-edge (2p_3/2_) around 853 eV and an L_2_-edge (2p_1/2_) around 870 eV due to the 2p spin-orbit coupling [100]. The structure of L_3_-edge and L_2_-edge multiplets is characteristic of high-spin pseudo-O_h_ Ni(II) complexes. In contrast to Cu(II), the L_3_-edge and L_2_-edge peaks are split into two features due to distortion of O_h_ geometry of the [Ni(NCS)_6_]^4−^ unit, which is observed mainly in the case of complexes **1** and **2** with thiocyanates as bridging ligands. The structure of **1** was formed as a result of bridging four thiocyanate ions in equatorial positions to copper centers. The other two ions in the *trans* position in [Ni(NCS)_6_]^4−^ remain non-bridging ligands. Complex **2** is the first example of bridging the thiocyanate ion as μ_1,3,3_-NCS that is not found in the {NiCu_2_} unit so far. In these complexes, the half-field e_g_ orbitals are split, and two peaks in the main part of the L-edge are observed with the energy of 1.0 eV and 1.2 eV for L_3_-edge and L_2_-edge features, respectively. The integrated intensity of the L_3_-edge transition multiplet decreases (Table 6) in the order: **1** → **2** → **3**, indicating that the average ligand character, and therefore the covalency of the unoccupied metal d-based orbitals, increases in the same order. Nickel L-edge energy did not change and was constant for all the complexes, confirming that nickel retained its configuration and coordination with the six thiocyanates, as shown in the crystallographic section.

### 3.6. Magnetism

**Figure 10:**
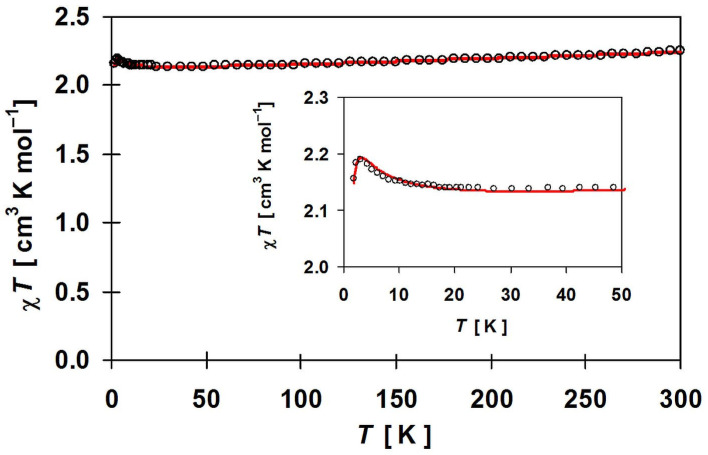
Thermal dependence of χ_M_T product for [{Cu(pn)_2_}_2_Ni(NCS)_6_]_n_∙2nH_2_O (**1**). Inset—the low-temperature range. The solid red line is the best fit for the model described in the text.

The plot of *χT* for **1** is given in Figure 10. In the whole measured range, it obeys the Curie–Weiss law with C = 2.104 cm^3^ mol^−1^ K and θ = 0.17 K. The value of *χT* at 298 K is equal to 2.25 cm^3^·mol^−1^·K (4.24 B.M.), which is slightly larger than 1.75 cm^3^·mol^−1^·K expected for two uncoupled Cu(II) ions (0.75; *S* = ^1^/_2_; g = 2.0) and one Ni(II) ion (1; S = 1; g = 2). The value of *χT* product decreases slightly with temperature, and at 50 K, reaches a minimum of 2.14 cm^3^·mol^−1^·K and then increases with the lowering temperature to the value 2.19 cm^3^·mol^−1^·K at 3.2 K. This is followed by a rapid decline of the *χT* product to 2.155 cm^3^·mol^−1^·K at 1.8 K. The increase in the value *χT* in the low-temperature range may be due to ferromagnetic interactions between metal ions through SCN^−^ ligands. This is supported by the positive Weiss constant. The small decrease up to 50 K can be caused by measurement inaccuracies due to small spins or temperature-independent paramagnetism, especially for Ni(II) ions [101,102], while the rapid lowering of the *χT* product at 1.8 K can be caused by antiferromagnetic interactions through hydrogen bonds and zero-field splitting for Ni(II).

**Figure 11:**
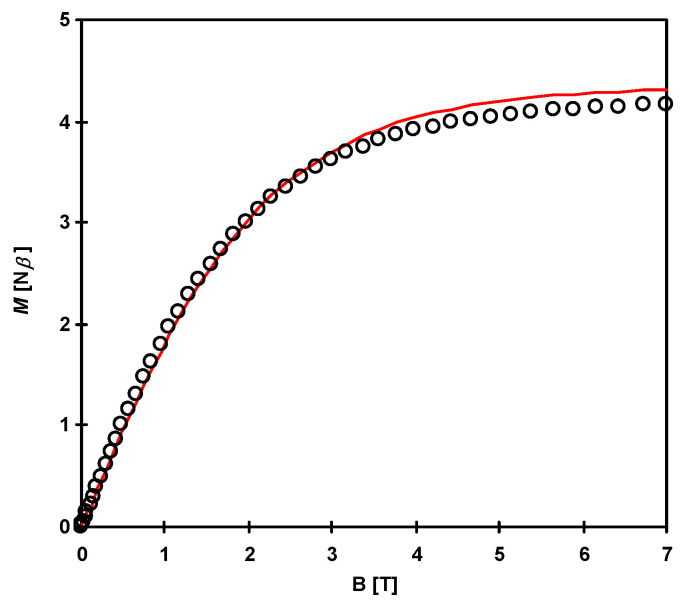
Field dependence of the magnetization for **1** at 1.8 K. The solid red line is the best fit for the model described in the text.

The magnetization curve at 1.8 K (Figure 11) shows no hysteresis loop. Magnetization at saturation equal 4.15 B.M. at 7 T corresponds to the ground state of metal ions with the total spin equal 2 for the {NiCu_2_} unit.

**Figure 12:**
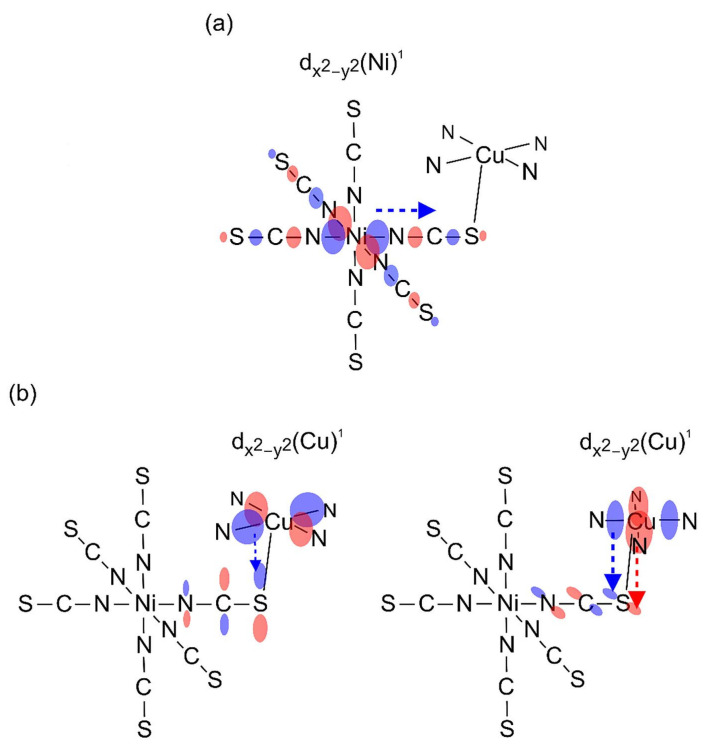
Schematic representation of spin density delocalization from the d_x_^2^_−y_^2^ orbital of Ni(II) onto the σ(SCN^−^) system (**a**) and from the d_x_^2^_−y_^2^ orbital of Cu(II) onto the π(SCN^−^) system (**b**) in **1**. The orthogonal orientation of the resultant natural magnetic orbital systems is in line with the weak ferromagnetic Ni(II)-Cu(II) interactions found from the fits of magnetic data.

Due to the 2D structure of **1**, it was possible to perform only approximate calculations of the magnetic parameters. Both the Cu-S bond lengths and the Cu-S-C angles of the two thiocyanate bridges, which are of key importance for the magnetic interactions, do not differ significantly (see above). Therefore, the {NiCu_2_} trimetallic unit was taken as a representative model for the analysis of magnetic properties. The Hamiltonian of the type *H* = −2*J*_CuNi_(S_Cu1_ S_Ni_ + S_Ni_ S_Cu2_) was applied. The *J*_CuNi_ is the Heisenberg exchange coupling constant between adjacent Cu(II)-Ni(II) ions. The magnetic data were fitted using the PHI program [103], taking into account also other parameters such as TIP, nickel(II) zero-field splitting and the molecular field. The best fit was obtained for the following parameters: *g*_av_ = 2.201, *J*_CuNi_ = 0.059 cm^−1^, z*J*’ = 0.019 cm^−1^, TIP = 461 × 10^−6^ cm^3^ mol^−1^ and *D*_Ni_ = 2.271 cm^−1^. We made various modifications to the model (Table S7, Supplementary Materials), which do not affect the sign of the *J*_CuNi_ exchange parameter, which is always positive with values ranging from 0.06 to 0.48 cm^−1^. Doubling of the considered interacting unit to the six-metal {Ni_2_Cu_4_} and introduction of an additional interaction between the trimetallic subunits leads to similar results. All the modeling tests carried out indicate that the interactions are indeed very weak but predominantly ferromagnetic. This can be explained as follows: The unpaired electrons occupy the e_g_ orbital of nickel(II) ion and d_x2–y2_ of copper(II) ion. Both d_z2_ and d_x2–y2_ Ni(II) magnetic orbitals overlap with the σ orbitals of the bridging thiocyanate ion. We also suggest that the spin density of d_x_^2^_–y_^2^ of Cu delocalizes on π(SCN^−^) systems, which leads to the orthogonal arrangement of both SOMO systems in line with effective weak ferromagnetic interactions derived from the fits of magnetic data (Figure 12). Somewhat stronger ferromagnetic interactions transmitted through thiocyanate ions have been reported for {Cu_2_Mn} analog, i.e., [{Cu(pn)_2_}_2_Mn(NCS)_6_]_n_∙2nH_2_O (*J* = 0.13 cm^−1^) [31].

**Figure 13:**
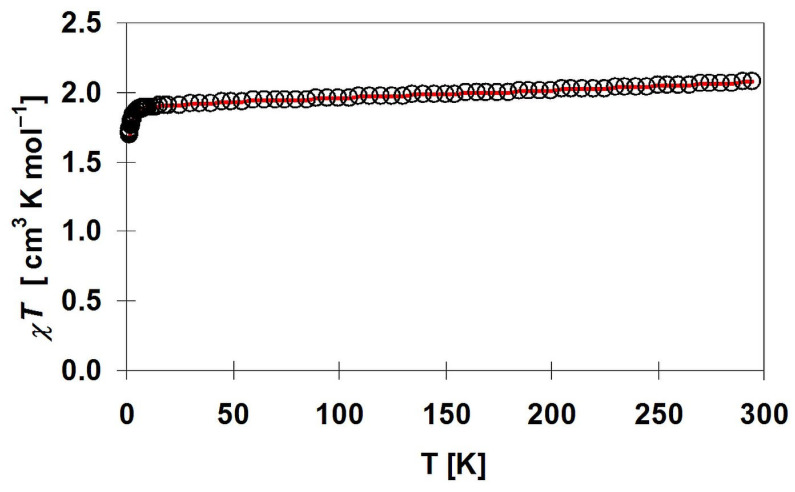
Thermal dependence of χ_M_T product for [{Cu^II^(trien)}_2_Ni(NCS)_6_Cu^I^(NCS)]_n_ (**2**). The solid red line is the best fit for the model described in the text.

The plot of *χT* for **2** is given in Figure 13. The *χT*(*T*) curve in the whole measured range obeys the Curie–Weiss law in the whole measured range with C = 2.049 cm^3^ mol^−1^ K and θ = −1.93 K. The value of *χT* at 295 K is equal to 2.07 cm^3^·mol^−1^·K (4.06 B.M.), which is slightly bigger than the value (1.75 cm^3^·mol^−1^·K) expected for two uncoupled Cu(II) ions (0.75; S = ^1^/_2_; g = 2.0) and one Ni(II) ion (1; S = 1; g = 2). Cu(I) ion is diamagnetic and gives no contribution to the magnetism except for the negligible contribution to the diamagnetic correction. The *χT* product decreases slowly with temperature to 10 K and then more rapidly reaches 1.69 cm^3^·mol^−1^·K at 1.8 K. The small decrease up to 10 K can be caused by measurement inaccuracies due to small spins or temperature-independent paramagnetism, especially for Ni(II) ions [101,102], while the rapid lowering of the *χT* product below 10 K can be caused by antiferromagnetic interactions through thiocyanate bridges and zero-field splitting for Ni(II). This is supported by the negative Weiss constant.

**Figure 14:**
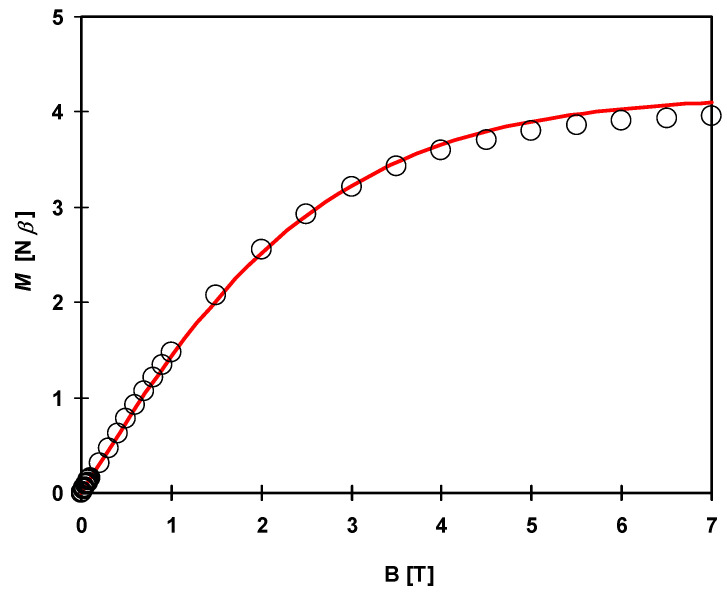
Field dependence of the magnetization for **2** at 2 K. The solid red line is the best fit for the model described in the text.

Due to structural reasons, the analysis of magnetic properties was based on the trimetallic {NiCu^II^_2_} unit, ignoring the bridges through [Cu(SCN)_3_(NCS)]^3−^ diamagnetic unit. The Hamiltonian of the type *H* = −2*J*_CuNi_(S_Cu1_ S_Ni_ + S_Ni_ S_Cu2_) –2*J*_CuCu_(S_Cu1_ S_Cu2_) was applied. The *J*_CuNi_ and *J*_CuCu_ are the Heisenberg exchange coupling constants between adjacent Cu(II)-Ni(II) and Cu(II)-Cu(II) ions, respectively. Magnetic data were fitted using the PHI program [103], taking into account also other parameters such as TIP, ZFS for Ni(II) ion and molecular field correction. The best fit (red lines in Figure 13 and Figure 14) was obtained for the following parameters: *g*_av_ = 2.085, *J*_CuNi_ = 0.057 cm^−1^, *J*_CuCu_ = –0.718 cm^−1^, z*J’* = 0.009 cm^−1^, TIP = 567 × 10^−6^ cm^3^ mol^−1^, *D*_Ni_ = 0.735 cm^−1^.

The magnetization curve at 2 K (Figure 14) shows no hysteresis loop. Saturation magnetization equals 3.96 B.M. at 7 T corresponds to the ground state of metal ions with the total spin equal 2 for the {NiCu_2_} unit.

**Figure 15:**
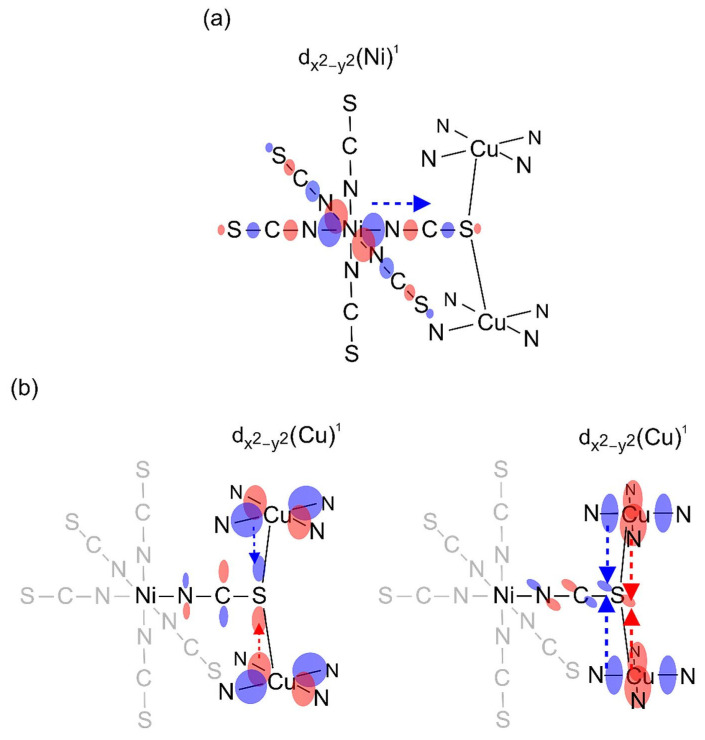
Schematic representation of spin density delocalization from the d_x_^2^−_y_^2^ orbital of Ni(II) onto the σ(SCN^−^) system (**a**) and from the d_x_^2^−_y_^2^ orbital of Cu(II) onto the π(SCN^−^) system (**b**) in **2**. The magnetic interaction Cu-Ni paths in **2** are similar to that of **1** depicted in Figure 12. However, due to significant overlap of natural magnetic orbitals of Cu(II) systems of end-on thiocyanato-bridged Cu(II) moieties (*d*_Cu-S_ *ca*. 2.36 Å) (**b**), the Cu-Cu antiferromagnetic interaction prevailed in **2**, as was shown by the fit of magnetic data.

The unpaired electrons are located on the e_g_ orbitals of nickel(II) ions and d_x_^2^_–y_^2^ orbital of copper(II) ions. However, in this case, octahedral nickel(II) and both square pyramidal copper(II) ions are linked by *µ*_1,3,3_-NCS anions in the manner that enables contacts between two d_z_^2^_–y_^2^ orbitals of both Cu complexes through the part of π(SCN^−^) system located on S atom (Figure 15). The magnetic interaction Cu-Ni paths are similar to **1**, but another antiferromagnetic Cu-Cu path appears with much shorter Cu-S distances (*ca.* 2.36 Å) compared to **1**, which thus dominates the scheme of magnetic exchange interactions.

The room temperature (293 K) effective magnetic moment of **3** is 4.91 B.M., which is slightly higher than the expected spin-only value for uncoupled four Cu(II) and one Ni(II) ions, i.e., 4.47 B.M. (4 × 0.5; S = 2; g = 2.0 and *S* = 1; g = 2). By taking into account that for both copper and nickel, the g factors usually are well over 2, we have an agreement for both g = 2.19.

## 4. Conclusions

We prepared [{Cu(pn)_2_}_2_Ni(NCS)_6_]_n_∙2nH_2_O (**1**), [{Cu^II^(trien)}_2_Ni(NCS)_6_Cu^I^(NCS)]_n_ (**2**) and [Cu(tren)(NCS)]_4_[Ni(NCS)_6_] (**3**) showing different coordination topologies—2D layer and 1D ladder together with a complex salt composed of discrete mononuclear species, respectively. In the structure of **1**, there are layers with paramagnetic centers connected by four thiocyanate anions. Hydrogen bond inspections revealed that the O7 water molecule plays a crucial role in crystal network formation, which is a pivot point for many interlayer hydrogen bonds. Compound **1** is a coordination polymer presenting a relatively scarce example of ferromagnetic coupling between nickel(II) and copper(II) ions. Compound **2** shows a rare ladder topology with rails and rungs formed by thiocyanate anions bridging arranged alternately by Cu(I) and Ni(II) ions, whereas [Cu^II^(trien)]^2+^ moieties are antennas decorating the ladder and acting as side chains connected to Ni(II) cations. Hence, in the description of the magnetic interaction, we took into account {NiCu^II^_2_} trinuclear units featuring *µ*_1,3,3_-NCS bridging mode that enables dominating antiferromagnetic interactions between the Cu moieties and separated by Cu(I) units. The unique ladders in this structure form a tightly packed system without solvent molecules and are connected by hydrogen bonds. The highest Cu:Ni ratio was reached in **3**, showing discrete mononuclear complexes forming columns connected by hydrogen bonds. In all three complexes, the topology was tailored due to the proper selection of the amine, and it affected the magnetic couplings. In (**1**), the ferromagnetic properties resulted from the spin density of Cu d_x_^2^_–y_^2^ delocalized onto π(SCN^−^) systems and hence, from the orthogonal arrangement of both SOMO systems in line with effective weak ferromagnetic interactions derived from the fits of magnetic data. In (**2**), the overall weak antiferromagnetic effect comes from two concurrent Cu-Ni (ferromagnetic) and Cu-Cu (antiferromagnetic) pathways, with the latter predominating. Compound (**3**), as a compound without a thiocyanato bridge, shows a spin-only magnetic moment. The differences in the topology also resulted in changes in Cu(II) coordination sphere geometry–6 coordinated 4+2 in **1**, square pyramid in **2** and trigonal bipyramid in **3**. Analysis of XAS proves that the average ligand character and the covalency of the unoccupied metal d-based orbitals for copper(II) and nickel(II) increase in the following order: **1** → **2** → **3**.

## Figures and Tables

**Table 1:** Crystal data and structure refinement for **1**, **2** and **3**.

Identification Code	1	2	3
Empirical formula	C_18_ H_44_ Cu_2_ N_14_ Ni O_2_ S_6_	C_19_ H_36_ Cu_3_ N_15_ Ni S_7_	C_34_ H_72_ Cu_4_ N_26_ Ni S_10_
Formula weight	866.82	948.38	1478.64
Temperature [K]	293(2)	293(2)	293(2)
Wavelength [Å]	0.71073	0.71073	0.71073
Crystal system, space group	monoclinic, C2/c (no 15)	orthorhombic, Pnma (62)	monoclinic, P21/c (14)
Unit cell dimensions [Å] and [°]	a = 15.2714(7) b = 16.7046(6) β = 98.419(4) c = 14.2730(7)	a = 16.6621(10) b = 12.5136(7) c = 17.6717(12)	a = 11.0628(9) b = 14.5681(11) β = 92.158(7) c = 19.3533(14)
Volume [Å^3^]	3601.9(3)	3684.6(4)	3116.8(4)
Z, Calculated density [Mg·m^−3^]	4, 1.598	4, 1.710	2, 1.576
Absorption coefficient [mm^−1^]	2.076	2.645	2.025
F(000)	1792	1928	1524
Crystal size [mm]	0.360 × 0.310 × 0.160	0.570 × 0.320 × 0.110	0.430 × 0.330 × 0.130
Theta range for data collection [°]	2.194 to 26.367	2.305 to 26.372	2.512 to 26.370
Limiting indices	−19 ≤ h ≤ 18 −20 ≤ k ≤ 20 −17 ≤ l ≤ 17	−20 ≤ h ≤ 20 −15 ≤ k ≤ 15 −22 ≤ l ≤ 20	−13 ≤ h ≤ 13 −18 ≤ k ≤ 17 −24 ≤ l ≤ 23
Reflections collected/unique	12068/3677 [R(int) = 0.0426]	25450/3949 [R(int) = 0.0255]	20016/6372 [R(int) = 0.0481]
Completeness [%] to theta [°]	25.242° 99.9%	25.242° 99.9%	25.242° 99.9%
Absorption correction	Numerical	Numerical	Numerical
Max. and min. transmission	0.732 and 0.522	0.760 and 0.314	0.779 and 0.476
Refinement method	Full-matrix least-squares on F^2^	Full-matrix least-squares on F^2^	Full-matrix least-squares on F^2^
Data/restraints/parameters	3677/3/219	3949/6/253	6372/0/367
Goodness-of-fit on F^2^	0.836	1.065	0.996
Final R Indices [I>2sigma(I)]	R1 ^a^ = 0.0396, wR2 ^b^ = 0.0936	R1 ^a^ = 0.0312, wR2 ^b^ = 0.0740	R1 ^a^ = 0.0386, wR2 ^b^ = 0.0902
R indices (all data)	R1 ^a^ = 0.0713, wR2 ^b^ = 0.1049	R1 ^a^ = 0.0375, wR2 ^b^ = 0.0778	R1 ^a^ = 0.0590, wR2 ^b^ = 0.1007
Largest diff. peak and hole [eÅ^−3^]	0.604 and −0.346	1.013 and −0.629	0.387 and −0.369

^a^ R1 = ∑‖*F_0_*|−|*F_c_*||/∑|*F_0_* | ^b^ wR2 = [∑w(*F_0_*^2^−*F_c_*^2^)^2^/∑(w(*F_0_*^2^)^2^)]^1/2^.

**Table 2:** Selected bond lengths [Å] for **1**.

Ni1-N5 ^i^	2.061(3)	Cu2-N2	2.003(3)
Ni1-N5	2.061(3)	Cu2-N12	2.010(3)
Ni1-N6^i^	2.073(3)	Cu2-N11	2.013(3)
Ni1-N6	2.073(3)	Cu2-N1	2.028(3)
Ni1-N4 ^i^	2.118(4)	Cu2-S5 ^i^	2.9883(11)
Ni1-N4	2.118(4)	Cu2-S6 ^ii^	3.0224(10)

^i^ −x, y,−z + 1/2 ^ii^ −0.5 + x,−0.5+y, z.

**Table 3:** Selected bond lengths [Å] for **2**.

Ni1-N5	2.048(3)	Cu2-N14	1.999(2)
Ni1-N2	2.084(2)	Cu2-N17	2.014(2)
Ni1-N2#1	2.084(2)	Cu2-N20	2.017(2)
Ni1-N1	2.082(3)	Cu2-N11	2.029(2)
Ni1-N4	2.091(3)	Cu2-S4	2.6422(4)
Ni1-N3	2.103(3)		
Cu3-N6	1.946(5)	Cu3-S2#3	2.3520(8)
Cu3-S2#2	2.3520(8)	Cu3-S3	2.3711(1)

#1 x, −y + 1/2,z #2 −x + 1, y, z + 1 #3 −x + 1, y + 1/2, −z + 1.

**Table 4:** Selected bond lengths [Å] for **3**.

Cu1-N5	1.950(3)	Cu3-N4	1.941(3)
Cu1-N27	2.041(3)	Cu3-N14	2.034(2)
Cu1-N24	2.042(2)	Cu3-N11	2.061(3)
Cu1-N21	2.042(3)	Cu3-N17	2.081(3)
Cu1-N30	2.128(3)	Cu3-N20	2.109(3)
Ni2-N2	2.075(3)	Ni2-N3	2.082(3)
Ni2-N2 ^i^	2.075(3)	Ni2-N1 ^i^	2.102(3)
Ni2-N3 ^i^	2.082(3)	Ni2-N1	2.102(3)

^i^ −x + 1, −y + 1, −z.

**Table 5:** Integrated intensities and energies of maximum for Cu L_3_-edge and L_2_-edge peaks for **1**, **2** and **3**.

Complex	Integrated L_3_-Edge Intensity 925–935	Integrated L_2_-Edge Intensity 945–955	Energy of Maximum L_3_-Edge eV	Energy of Maximum L_2_-Edge eV
[{Cu(pn)_2_}_2_Ni(NCS)_6_]_n_∙2nH_2_O (**1**)	10.5	1.8	931.6	951.4
[{Cu^II^(trien)}_2_Ni(NCS)_6_Cu^I^(NCS)]_n_ (**2**)	9.2	2.0	931.4	951.2
[Cu(tren)(NCS)]_4_[Ni(NCS)_6_] (**3**)	7.1	3.2	931.2	951.2

**Table 6:** Integrated intensities and energies of maximum for Ni L_3_-edge and L_2_-edge peaks for **1**, **2** and **3**.

Complex	Integrated L_3_-Edge Intensity 850–860	Integrated L_2_-Edge Intensity 865–875	Energy of Maximum L_3_-Edge eV	Energy of Maximum L_2_-Edge eV
[{Cu(pn)_2_}_2_Ni(NCS)_6_]_n_∙2nH_2_O (**1**)	11.9	4.2	853.4	870.6
			855.4	871.8
[{Cu^II^(trien)}_2_Ni(NCS)_6_Cu^I^(NCS)]_n_ (**2**)	10.2	3.9	853.4	870.4
			855.4	871.6
[Cu(tren)(NCS)]_4_[Ni(NCS)_6_] (**3**)	10.1	4.5	853.4	870.6
			855.4	871.8

## Data Availability

Data are contained within the article, Supplementary Materials and CCDC database (deposited structures).

## Supplementary Materials

The supporting information can be downloaded at: https://www.mdpi.com/article/10.3390/ma16020731/s1. Supplementary Materials contain data concerning valence angles (Tables S1, S3 and S5) and intermolecular hydrogen bonds (Tables S2, S4 and S6) for (**1**), (**2**) and (**3**), respectively, magnetic data (Table S7), figures presenting packing of (**1**) (Figure S1) and layer arrangement for (**1**) (Figure S2), Analysis of intermolecular interactions mapped onto Hirshfeld surface and given as a fingerprint in **3** (Figure S3), XAS spectra of (**2**) (Figure S4) and (**3**) (Figure S5) and normaliyed N K+edge absorption spectra for (**1**), (**2**) and (**3**) (Figure S6).

## References

[1] Van der Laan G., Figueroa A.I. (2014). X-ray magnetic circular dichroism—A versatile tool to study magnetism. Coord. Chem. Rev..

[2] Thorarinsdottir A.E., Harris T.D. (2020). Metal-Organic Framework Magnets. Chem. Rev..

[3] Mingabudinova L.R., Vinogradov V.V., Milichko V.A., Hey-Hawkin E., Vinogradov A.V. (2015). Metal–organic frameworks as competitive materials for non-linear optics. Chem. Soc. Rev..

[4] Cui Y., Yue Y., Qian G., Chen B. (2012). Luminescent functional metal-organic frameworks. Chem. Rev..

[5] Pardo R., Zayat M., Levy D. (2010). Photochromic organic–inorganic hybrid materials. Chem. Soc. Rev..

[6] Sodhi R.K., Paul S. (2019). Metal Complexes in Medicine: An Overview and Update from Drug Design Perspective. Cancer Ther. Oncol. Int. J..

[7] Saini P., Sonika, Singh G., Kaur G., Singh J., Singh H. (2021). Robust and Versatile Cu(I) metal frameworks as potential catalysts for azide-alkyne cycloaddition reactions: Review. Mol. Catal..

[8] Kalra P., Kaur R., Singh G., Singh H., Singh G., Pawan, Kaur G., Singh J. (2021). Metals as “Click” catalysts for alkyne-azide cycloaddition reactions: An overview. J. Organomet. Chem..

[9] Nasibipour M., Safaei E., Wrzeszcz G., Wojtczak A. (2020). Tuning of the redox potential and catalytic activity of a new Cu(II) complex by o-iminobenzosemiquinone as an electron-reservoir ligand†. New J. Chem..

[10] Gispert J.R. (2008). Coordination Chemistry.

[11] Kinzel N.W., Demirbas D., Bill E., Weyhermüller T., Werlé C., Kaeffer N., Leitner W. (2021). Systematic Variation of 3d Metal Centers in a Redox-Innocent Ligand Environment: Structures, Electrochemical Properties, and Carbon Dioxide Activation. Inorg. Chem..

[12] Muzioł T.M., Tereba N., Podgajny R., Kędziera D., Wrzeszcz G. (2019). Solvent-assisted structural conversion involving bimetallic complexes based on the tris(oxalato)ferrate(III) unit with the green → blue → red crystal color sequence. Dalton Trans..

[13] Zhou Y., Zhang Y.-L., Zhang Q., Yang S.-Y., Wei X.-Q., Tian Z., Shao D. (2022). Supramolecular porous frameworks of two Ni(II) coordination polymers with varying structures, porosities, and magnetic properties. Polyhedron.

[14] Zhou Y., Xiang H., Zhu J.-Y., Shi L., You W.-J., Wei X.-Q., Tian Z., Shao D. (2022). Synthesis, structure, magnetism and proton conductivity of a cyanide-bridged Ni^II^Co^III^ framework. Polyhedron.

[15] Espallargas G.M., Coronado E. (2018). Magnetic functionalities in MOFs: From the framework to the pore. Chem. Soc. Rev..

[16] Clemente-León M., Coronado E., Martí-Gastaldo C., Romero F.M. (2011). Multifunctionality in hybrid magnetic materials based on bimetallic oxalate complexes. Chem. Soc. Rev..

[17] Ko M., Mendecki L., Mirica K.A. (2018). Conductive two-dimensional metal–organic frameworks as multifunctional materials. Chem. Commun..

[18] Zhang H., Wang X., Zhang K., Teo B.K. (1990). Molecular and crystal engineering of a new class of inorganic cadmium-thiocyanate polymers with host–guest complexes as organic spacers, controllers, and templates. Coord. Chem. Rev..

[19] González R., Acosta A., Chiozzone R., Kremer C., Armentano D., De Munno G., Julve M., Lloret F., Faus J. (2012). New Family of Thiocyanate-Bridged Re(IV)-SCN-M(II) (M = Ni, Co, Fe, and Mn) Heterobimetallic Compounds: Synthesis, Crystal Structure, and Magnetic Properties. Inorg. Chem..

[20] Bieńko A., Kłak J., Mroziński J., Boča R., Brüdgam I., Hartl H. (2007). Trinuclear thiocyanate-bridged compounds of the type [ML]_2_[Mn(NCS)_4_](ClO_4_)_2_ (where M = Cu(II), Ni(II); L = *N*-*dl*-5,7,7,12,14,14-hexamethyl-1,4,8,11-tetraazacyclotetradeca-4,11-diene). Dalton Trans..

[21] Quan Y.-P., Yin P., Han N.-N., Yang A.-H., Gao H.-L., Cui J.-Z., Shi W., Cheng P. (2009). Novel hetero-polynuclear metal complexes (CuL)_3_[Mn(NCS)_5_]_2_ and (NiL)_3_[Mn(NCS)_5_]_2_ containing trigonal bipyramidal geometric [Mn(NCS)_5_]^3–^ as bridging ligand. Inorg. Chem. Comm..

[22] Bose D., Mostafa G., Bailey Walsh R.D., Zaworotko M.J., Ghosh B.K. (2006). Bimetallic complex of the type [Cu(tren)(NCS)]_4_[Mn(NCS)_6_]: A hydrogen bonded network structure. Polyhedron.

[23] Kobayashi M., Savard D., Geisheimer A.R., Sakai K., Leznoff D.B. (2013). Heterobimetallic Coordination Polymers Based on the [Pt(SCN)_4_]^2−^ and [Pt(SeCN)_4_]^2−^ Building Blocks. Inorg. Chem..

[24] Khandar A.A., Klein A., Bakhtiari A., Mahjoub A.R., Pohl R.W.H. (2011). One-dimensional ladder like and two-dimensional polymorphs of heterometallic thiocyanate bridged copper(II) and mercury(II) coordination polymer: Syntheses, structural, vibration, luminescence and EPR studies. Inorg. Chim. Acta.

[25] Pryma O.V., Petrusenko S.R., Kokozay V.N., Skelton B.W., Shishkin O.V., Teplytska T.S. (2003). A Facile Direct Synthesis of Bimetallic Cu^II^Zn^II^ Complexes with Ethylenediamine Revealing Different Types of Chain Crystal Structures. Eur. J. Inorg. Chem..

[26] Wrzeszcz G., Muzioł T.M., Tereba N. (2015). Synthesis, characterization and crystal structure of a 1D thiocyanato bridged [Cu(en)_2_Zn(NCS)_4_]∙H_2_O. Comparison of the three structures with the same [Cu(en)_2_Zn(NCS)_4_] unit—different in structural terms. J. Mol. Struct..

[27] Tereba N., Muzioł T.M., Podgajny R., Wrzeszcz G. (2019). Influence of the Substituted Ethylenediamine Ligand on the Structure and Properties of [Cu(diamine)_2_Zn(NCS)_4_]⋅Solv. Compounds. Crystals.

[28] Mousavi M., Duhayon C., Bretosh K., Bereau V., Sutter J.-P. (2020). Molybdenum(III) Thiocyanate- and Selenocyanate-Based One-Dimensional Heteronuclear Polymers: Coordination Affinity-Controlled Assemblage of Mixed Spin and Mixed Valence Derivatives with Ni(II) and Co(II/III). Inorg. Chem..

[29] Burla M.C., Chiari B., Cinti A., Piovesana O. (1995). Structure and Magnetism of a New 2-D Bimetallic Compound of Mn(II) and Cu(II). Mol. Cryst. Liq. Cryst..

[30] Shen L., Xu Y.-Z. (2001). Structure and magnetic properties of a novel two-dimensional thiocyanato-bridged heterometallic polymer {Cu(en)_2_[Ni(en)(SCN)_3_]_2_}_n_. J. Chem. Soc. Dalton Trans..

[31] Shi J.-M., Xu W., Zhao B., Cheng P., Liao D.-Z., Chen X.-Y. (2005). A 2D Thiocyanato-Bridged Copper(II)-Manganese(II) Bimetallic Coordination Polymer with Ferromagnetic Interactions. Eur. J. Inorg. Chem..

[32] Mousavi M., Béreau V., Duhayon C., Guionneauc P., Sutter J.-P. (2012). First magnets based on thiocyanato-bridges. Chem. Commun..

[33] Xie K.-P., Xu W.-J., He C.-T., Huang B., Du Z.-Y., Su Y.-J., Zhang W.-X., Chena X.-M. (2016). Order–disorder phase transition in the first thiocyanate-bridged double perovskite-type coordination polymer: [NH_4_]_2_[NiCd(SCN)_6_]. CrystEngComm.

[34] Cliffe M.J., Keyzer E.N., Bond A.D., Astle M.A., Greya C.P. (2020). The structures of ordered defects in thiocyanate analogues of Prussian Blue. Chem. Sci..

[35] Maity D., Chattopadhyay S., Ghosh A., Drew M.G.B., Mukhopadhyay G. (2011). Syntheses, characterization and X-ray crystal structures of a mono- and a penta-nuclear nickel(II) complex with oximato Schiff base ligands. Inorg. Chim. Acta.

[36] Shurdha E., Moore C.E., Rheingold A.L., Lapidus S.H., Stephens P.W., Arif A.M., Miller J.S. (2013). First Row Transition Metal(II) Thiocyanate Complexes, and Formation of 1-, 2-, and 3-Dimensional Extended Network Structures of M(NCS)_2_(Solvent)_2_ (M = Cr, Mn, Co) Composition. Inorg. Chem..

[37] Hofffman D.W., Wood J.S. (1982). Tetramethylammonium hexaisothiocyanatonickelate(II) [(CH_3_)_4_]_4_Ni(NCS)_6_. Cryst. Struct. Commun..

[38] Kruger P.E., McKee V. (1996). Tetrakis(triethylammonium) Hexakis(isothiocyanato-*N*)nickel(II). Acta Crystallogr. Sect. C Cryst. Struct. Commun..

[39] Vijayakanth T., Ram F., Praveenkumar B., Shanmuganathan K., Boomishankar R. (2020). Piezoelectric Energy Harvesting from a Ferroelectric Hybrid Salt [Ph_3_MeP]_4_[Ni(NCS)_6_] Embedded in a Polymer Matrix. Angew. Chem. Int. Ed..

[40] Bi J.-H., Bi W.-T., Huang Z.-X., Hu N.-L. (2009). Synthesis and Crystal Structure of [(phen)_3_Co]_2_⋅Ni(SCN)_6_. Asian J. Chem..

[41] Song M.-P., Li L.-K., Wu B.-L., Niu Y.-Y. (2007). Bis(5,8-diazoniadispiro[4.2.4.2]tetradecane) hexakis(thiocyanato-*κN*)-nickelate(II) dihydrate. Acta Crystallogr. Sect. E Struct. Rep. Online.

[42] Liu J.-Y., Zhang S.-Y., Zeng Y., Shu X., Du Z.-Y., He C.-T., Zhang W.-X., Chen X.-M. (2018). Molecular Dynamics, Phase Transition and Frequency-Tuned Dielectric Switch of an Ionic Co-Crystal. Angew. Chem. Int. Ed..

[43] Kushch N.D., Bardin A.A., Buravov L.I., Glushakova N.M., Shilov G.V., Dmitriev A.I., Morgunov R.B., Kulikov A.V. (2014). Synthesis particularities, structure and properties of the radical cationsalts ω-(BEDT-TTF)_5_M(SCN)_6_·C_2_H_5_OH, M = Mn, Ni. Synth. Met..

[44] López Lago E., Seijas J.A., de Pedro I., Rodríguez Fernández J., Vázquez-Tato M.P., González J.A., Rilo E., Segade L., Cabeza O., Rodríguez Fernández C.D. (2018). Structural and physical properties of a new reversible and continuous thermochromic ionic liquid in a wide temperature interval: [BMIM]_4_[Ni(NCS)_6_]. New J. Chem..

[45] Brinzari T.V., Tian C., Halder G.J., Musfeldt J.L., Whangbo M.-H., Schlueter J.A. (2009). Properties and Structural Phase Transition in Penta- And Hexacoordinate Isothiocyanato Ni(II) Compounds. Inorg. Chem..

[46] Vicente R., Escuer A., Solanas X., Font-Bardía M. (1996). Aqueous syntheses and crystal structures of the hexa- and pentacoordinated nickel(II) isothiocyanato derivatives of bulky triamines: (H_2_Et_5_dien)_2_[Ni(NCS)_6_], [Ni(Me_5_dien)(NCS)_2_] and [(Me_4_Etdien)(NCS)_2_]. Inorg. Chim. Acta.

[47] Jia Z.-H., Liu J.-Y., Liu D.-X., Zhang S.-Y., Du Z.-Y., He C.-T., Zhang W.-X., Chenc X.-M. (2021). Four-step thermosensitive dielectric response arising from motionable low-symmetry ammonium confined in deformable supramolecular cages. J. Mater. Chem. C.

[48] Tomkiewicz A., Kłak J., Mroziński J. (2004). Bimetallic complexes with macrocyclic ligands. Variation of magnetic exchange interactions in some heteronuclear thiocyanato-bridged compounds. Mat. Sci. Pol..

[49] Rad A.R.S., Khoshgouei M.B., Rezvani A.R. (2011). Water gas shift reaction over Zn–Ni/SiO_2_ catalyst prepared from [Zn(H_2_O)_6_]_2_[Ni(NCS)_6_]·H_2_O/SiO_2_ precursor. J. Mol. Catal. A Chem..

[50] Laure B., Tran L.-T., Luneau D., Reber C. (2003). Crystal structures, magnetic properties, and absorption spectra of nickel(II) thiocyanato complexes: A comparison of different coordination geometries. Can. J. Chem..

[51] Cabeza O., Varela L.M., Rilo E., Segade L., Domínguez-Pérez M., Ausín D., de Pedro I., Rodríguez Fernández J., González J., Vazquez-Tato M.P. (2019). Synthesis, microstructure and volumetry of novel metal thiocyanate ionic liquids with [BMIM] cation. J. Mol. Liq..

[52] House J.E., Marquardt L.A. (1989). Synthesis and thermal decomposition of piperidinium hexathiocyanatonickelate(II). Thermochim. Acta.

[53] Wang C.-F., Zhu Z.-Y., Zhou X.-G., Weng L.-H., Shen Q.-S., Yan Y.-G. (2006). Polymorphism and reactivity of [Ni(pyridine)_4_(NCS)_2_]: Two new supramolecular isomers and one macro-ionic derivative [(N-Methylpyridinium)_n_]_2_^2n+^·[Ni(μ_1,3_–SCN)_2_(NCS)_2_]_n_^2n−^. Inorg. Chem. Commun..

[54] Ju Z.-F., Yao Q.-X., Wu W., Zhang J. (2008). Strong electron-accepting methylviologen dication confined in magnetic hosts: Synthesis, structural characterization, charge-transfer and magnetic properties of {(MV)_2_[Ni(SCN)_5_]·Cl·2H_2_O}_n_ and {(MV)[M(N_3_)_2_(SCN)_2_]}_n_ (M = Mn, Co). Dalton Trans..

[55] Fleck M. (2004). Thiocyanates of nickel and caesium: Cs**_2_**NiAg**_2_**(SCN)**_6_**·2H**_2_**O and CsNi(SCN)**_3_**. Acta Crystallogr. Sect. C Cryst. Struct. Commun..

[56] Chekhlov A.N. (2008). (18-crown-6)potassium tris(thiocyanato)nickelate(II): Synthesis and crystal structure. Russ. J. Coord. Chem..

[57] Dobrzańska L., Wrzeszcz G., Grodzicki A., Rozpłoch F. (2000). Synthesis and Characterization of Thiocyanato-Bridged Heteropolynuclear Chromium(III)–Copper(II) Complexes. Pol. J. Chem..

[58] Dobrzańska L., Wrzeszcz G., Grodzicki A., Rozpłoch F. (2000). Synthesis and Properties of Thiocyanato-Bridged Chromium(III)-Copper(II) Hydroxo Complexes. Pol. J. Chem..

[59] Dobrzańska L., Wrzeszcz G., Grodzicki A., Rozpłoch F. (2001). Synthesis and Properties of New Bimetallic Complexes of General Formula: [Ni(diamine_2_]_3_[Cr(NCS)_6_]_2_·nH_2_O. Pol. J. Chem..

[60] Wrzeszcz G., Dobrzańska L., Grodzicki A., Wojtczak A. (2002). Magnetostructural characterisation of the first bimetallic assemblies derived from the anionic building block [Cr(NCS)_6_]^3−^, [M(en)_3_]*_n_*[{M(en)_2_-µ-SCN-Cr(NCS)_4_-µ-NCS}_2*n*_] with M = Ni(II), Zn(II). J. Chem. Soc. Dalton Trans..

[61] Wrzeszcz G., Dobrzańska L., Grodzicki A., Rozpłoch F. (2003). Synthesis and Characterization of New Thiocyanato Bridged Complexes with the General Formula [ML_n_]_3_[Cr(NCS)_6_]_2_∙mH_2_O, where M = Cu(II), Ni(II), Co(II); L = Various Substituted Imidazoles. Pol. J. Chem..

[62] Dobrzańska L., Wrzeszcz G., Grodzicki A., Rozpłoch F. (2001). Synthesis, Spectroscopy and Magnetism of New µ-Thiocyanato Polynuclear Copper(II)–Chromium(III) Complexes. Pol. J. Chem..

[63] Wrzeszcz G., Dobrzańska L. (2003). Magnetic and Thermal Properties of New Thiocyanato Bridged Complexes of the Type [M(diamine)_2_]_3_[Cr(NCS)_6_]_2_∙nH_2_O, where M = Cu(II), Ni(II). Pol. J. Chem..

[64] Wrzeszcz G. (2003). Synthesis and Characterization of New Thiocyanato Bridged Heterobimetallic Complexes with the General Formula: [Cu(diamine)_2_]_3_[Cr(NCS)_6_]_2_∙nH_2_O. Pol. J. Chem..

[65] Wrzeszcz G., Grzebielucha T. (2009). Synthesis and Properties of New Bimetallic Complexes of General Formula: [Cu(diamine)_2_][Cr(NCS)_4_(NH_3_)_2_]_2_. Pol. J. Chem..

[66] Baker M.L., Mara M.W., Yan J.J., Hodgson K.O., Hedman B., Solomon E.I. (2017). K- and L-edge X-ray absorption spectroscopy (XAS) and resonant inelastic X-ray scattering (RIXS) determination of differential orbital covalency (DOC) of transition metal sites. Coord. Chem. Rev..

[67] Bain G.A., Berry J.F. (2008). Diamagnetic corrections and Pascal’s constants. J. Chem. Educ..

[68] Figgis N., Nyholm R.S. (1958). A Convinient Solid for Calibration of the Gouy Magnetic Susceptibility Apparatus. J. Chem. Soc..

[69] (2000). CrysAlis RED and CrysAlis CCD.

[70] Sheldrick G.M. (2015). Crystal Structure Refinement with SHELXL. Acta Crystallogr. Sect. C Struct. Chem. Acta Crystallogr..

[71] Brandenburg K. (2001). DIAMOND, Release 2.1e.

[72] Farrugia L.J. (2012). WinGX and ORTEP for Windows: Un update. J. Appl. Crystallogr..

[73] Nakamoto K. (2009). Infrared and Raman Spectra of Inorganic and Coordination Compounds, pt. B.

[74] Buckley R.C., Wardeska J.G. (1972). Linkage isomerism of bridging thiocyanate in binuclear complexes. Inorg. Chem..

[75] Baer C., Pike J. (2010). Infrared Spectroscopic Analysis of Linkage Isomerism in Metal−Thiocyanate Complexes. J. Chem. Educ..

[76] Guo G., Xu Y., Cao J., Hu C. (2012). The {V_4_Nb_6_O_30_} Cluster: A New Type of Vanadoniobate Anion Structure. Chem. Eur. J..

[77] Triščíková L., Chomič J., Abboud K.A., Park J.-H., Meisel M.W., Černák J. (2004). Trinuclear Cu(pn)_2_Ag_2_(CN)_4_: Preparation, crystal structure and properties (pn=1,2-diaminopropane). Inorg. Chim. Acta.

[78] Mistry S., Natarajan S. (2018). Synthesis, structures and magnetic studies of new copper-azides. Inorg. Chim. Acta.

[79] Mroziński J., Kłak J., Kruszyński R. (2008). Crystal structure and magnetic properties of the 1D bimetallic thiocyanate bridged compound: {(CuL_1_)[Co(NCS)_4_]}(L_1_ = N-rac-5,12-Me_2_-[14]-4,11-dieneN_4_). Polyhedron.

[80] Blatov V.A., Shevchenko A.P., Proserpio D.M. (2014). Applied Topological Analysis of Crystal Structures with the ProgramPackage ToposPro. Cryst. Growth Des..

[81] Bieńko A., Kłak J., Mroziński J., Domagała S., Korybut-Daszkiewicz B., Woźniak K. (2007). Magnetism and crystal structures of Cu^II^Mn^II^ and Cu^II^Ni^II^ ordered bimetallic chains. Polyhedron.

[82] Ribas J., Diaz C., Costa R., Tercero J., Solans X., Font-Bardía M., Stoeckli-Evans H. (1998). Synthesis and Magnetic Properties of Four New (Cu−Ni)_2_ Tetranuclear Complexes of General Formula [Cu(oxpn)Ni(μ-NCS)(H_2_O)(aa)]_2_(X)_2_ (oxpn = *N,N*‘-Bis(3-aminopropyl)oxamide; aa = Bidentate Amine; X = ClO_4_^-^or PF_6_^-^). Ferro- and Antiferromagnetic Alternation. Inorg. Chem..

[83] Yang L., Powell D.R., Houser R.P. (2007). Structural variation in copper(I) complexes with pyridylmethylamide ligands: Structural analysis with a new four-coordinate geometry index, τ_4_. Dalton Trans..

[84] Okuniewski A., Rosiak D., Chojnacki J., Becker B. (2015). Coordination polymers and molecular structures among complexes of mercury(II) halides with selected 1-benzoylthioureas. Polyhedron.

[85] Addison A.W., Nageswara Rao T., Reedijk J., van Rijn J., Verschoor G.C. (1984). Synthesis, structure, and spectroscopic properties of copper(**II**) compounds containing nitrogen–sulphur donor ligands; the crystal and molecular structure of aqua[1,7-bis(***N***-methylbenzimidazol-2′-yl)-2,6-dithiaheptane]copper(II) perchlorate. J. Chem. Soc. Dalton Trans..

[86] Marongiu G., Lingafelter E.C., Paoletti P. (1969). Crystal structure of thiocyanatotriethylenetetraminecopper(II) thiocyanate. Inorg. Chem..

[87] Marongiu G., Cannas M. (1979). Crystal structures of thiocyanate polyamine copper(II) complexes. Part 7. (3,6-Diazaoctane-1,8-diamine)isothiocyanatocopper(II) perchlorate: A disordered structure. J. Chem. Soc. Dalton Trans..

[88] Sharma M., Ganeshpandian M., Majumder M., Tamilarasan A., Sharma M., Mukhopadhyay R., Islam N.S., Palaniandavar M. (2020). Octahedral copper(**ii**)-diimine complexes of triethylenetetramine: Effect of stereochemical fluxionality and ligand hydrophobicity on Cu^II^/Cu^I^ redox, DNA binding and cleavage, cytotoxicity and apoptosis-inducing ability. Dalton Trans..

[89] Tian C.-B., Li Z.-H., Lin J.-D., Wu S.-T., Du S.-W., Lin P. (2010). Cluster-Based Cu^II^–Azide Polymers: Synthesis, Structure, Magnetic Properties, and Effect of Polyamines on Crystal Structures. Eur. J. Inorg. Chem..

[90] Kitajgorodskij A.I. (1973). Molecular Crystals and Molecules.

[91] Woollard-Shore J.G., Holland J.P., Jones M.W., Dilworth J.R. (2010). Nitrite reduction by copper complexes. Dalton Trans..

[92] Pérez-Toro I., Domínguez-Martín A., Choquesillo-Lazarte D., García-Rubiño M.E., González-Pérez J.M., Castiñeiras A., Bauzá A., Frontera A., Niclós-Gutiérrez J. (2018). Copper(II) polyamine chelates as efficient receptors for acyclovir: Syntheses, crystal structures and dft study. Polyhedron.

[93] Herrera J.M., Marvaud V., Verdaguer M., Marrot J., Kalisz M., Mathonière C. (2004). Reversible Photoinduced Magnetic Properties in the Heptanuclear Complex [Mo^IV^(CN)_2_(CN-CuL)_6_]^8+^: A Photomagnetic High-Spin Molecule. Angew. Chem. Int. Ed..

[94] Gu Z.-G., Na J.-J., Wang B.-X., Xiao H.-P., Li Z. (2011). Novel copper-azido magnetic molecular tapes: Syntheses, structures, and magnetic properties. CrystEngComm.

[95] Spackman M.A., Jayatilaka D. (2009). Hirshfeld surface analysis. CrystEngComm.

[96] Spackman M.A., McKinnon J.J. (2002). Fingerprinting intermolecular interactions in molecular crystals. CrystEngComm.

[97] de Groot F.M.F. (1995). Differences between L_3_ and L_2_ X-ray absorption spectra. Phys. B Condens. Matter.

[98] Hocking R.K., DeBeer George S., Raymond K.N., Hodgson K.O., Hedman B., Solomon E.I. (2010). Fe L-Edge X-ray Absorption Spectroscopy Determination of Differential Orbital Covalency of Siderophore Model Compounds: Electronic Structure Contributions to High Stability Constants. J. Am. Chem. Soc..

[99] Hocking R.K., Wasinger E.C., de Groot F.M.F., Hodgson K.O., Hedman B., Solomon E.I. (2006). Fe L-Edge XAS Studies of K_4_[Fe(CN)_6_] and K_3_[Fe(CN)_6_]:  A Direct Probe of Back-Bonding. J. Am. Chem. Soc..

[100] van Elp J., Peng G., Zhou Z.H., Adams M.W.W., Baidya N., Mascharak P.K., Cramer S.P. (1995). Nickel L-Edge X-ray Absorption Spectroscopy of *Pyrococcus furiosus* Hydrogenase. Inorg. Chem..

[101] Nemec I., Herchel R., Boča R., Svoboda I., Trávníček Z., Dlháň L., Matelková K., Fuess H. (2011). Heterobimetallic assemblies of Ni(II) complexes with a tetradentate amine ligand and diamagnetic cyanidometallates. Inorg. Chim. Acta.

[102] Baran P., Boča M., Boča R., Krutošíková A., Miklovič J., Pelikán J., Titiš J. (2005). Structural characterization, spectral and magnetic properties of isothiocyanate nickel(II) complexes with furopyridine derivatives. Polyhedron.

[103] Chilton N.F., Anderson R.P., Turner L.D., Soncini A., Murray K.S. (2013). PHI: A Powerful New Program for the Analysis of Anisotropic Monomeric and Exchange-Coupled Polynuclear d- and f-Block Complexes. J. Comput. Chem..

