# Supplementary material for: Structural Diversity, XAS and Magnetism of Copper(II)-Nickel(II) Heterometallic Complexes Based on the [Ni(NCS)6]4− Unit
Source: Materials (Basel). 2023 Jan 11;16(2):731. doi: 10.3390/ma16020731 (PMC9861906; doi:10.3390/ma16020731)
Supplement: Supplementary file 1 [file materials-16-00731-s001.zip › materials-2139986-supplementary.pdf]

Supporting Information

Structural Diversity, XAS and Magnetism of Copper(II)-nickel(II) Heterometallic Complexes Based on the $[Ni(NCS)_6]^{4-}$ Unit

Natalia Tereba [1], Tadeusz M. Muzioł [1,*], Joanna Wiśniewska [1,*], Robert Podgajny [2], Alina Bieńko [3], Grzegorz Wrzeszcz [1,*]

1 Faculty of Chemistry, Nicolaus Copernicus University in Toruń, Gagarina 7, 87-100 Torun, Poland; natalia.tereba@wp.pl
2 Faculty of Chemistry, Jagiellonian University, Gronostajowa 2, 30-387 Cracow, Poland; robert.podgajny@uj.edu.pl
3 Faculty of Chemistry, University of Wroclaw, Joliot-Curie 14, 50-383 Wroclaw, Poland; alina.bienko@chem.uni.wroc.pl
* Correspondence: tmuziol@umk.pl (T.M.M.); wisnia@umk.pl (J.W.); wrzeszcz@umk.pl (G.W.)

Table of content **Page**

Table S1. Selected valence angles [°] for **1**. S2

Table S2**.** Intermolecular hydrogen bonds in the crystal network of $[\{Cu(pn)_2\}_2Ni(NCS)_6]_n{\cdot}2nH_2O$ (**1**). S2

Table S3. Selected valence angles [°] for **2**. S3

Table S4. Intermolecular hydrogen bonds in the crystal network of $[\{Cu^{II}(trien)\}_2Ni(NCS)_6Cu^{I}(NCS)]_n$ (**2**). S3

Table S5. Selected valence angles [°] for **3**. S4

Table S6. Intermolecular hydrogen bonds in the crystal network of $[Cu(tren)(NCS)]_4[Ni(NCS)_6]$ (**3**). S4

Table S7. Magnetic parameters for **1** S5

Figure S1. Packing of $[\{Cu(pn)_2\}_2Ni(NCS)_6]_n{\cdot}2nH_2O$ (**1**) along *a* axis shows robust *ab* layers S6

Figure S2. The arrangement of layers in **1** S6

Figure S3. Analysis of intermolecular interactions mapped onto Hirshfeld surface and given as a fingerprint in **3** S7-S9

Figure S4. The XAS spectra of $[\{Cu^{II}(trien)\}_2Ni(NCS)_6Cu^{I}(NCS)]_n$ (**2**) S10

Figure S5. The XAS spectra of $[Cu(tren)(NCS)]_4[Ni(NCS)_6]$ (**3**) S10

Figure S6. Normalized N K-edge absorption spectra for $[\{Cu(pn)_2\}_2Ni(NCS)_6]_n{\cdot}2nH_2O$ (**1**), $[\{Cu^{II}(trien)\}_2Ni(NCS)_6Cu^{I}(NCS)]_n$ (**2**) and $[Cu(tren)(NCS)]_4[Ni(NCS)_6]$ (**3**). S11

Table S1. Selected valence angles [°] for **1**.

N5^{i}-Ni1-N5	88.28(19)	N2-Cu2-N12	177.08(13)
N5^{i}-Ni1-N6^{i}	92.16(14)	N2-Cu2-N11	95.37(11)
N5-Ni1-N6^{i}	179.40(12)	N12-Cu2-N11	84.56(11)
N5^{i}-Ni1-N6	179.40(12)	N2-Cu2-N1	84.58(11)
N5-Ni1-N6	92.16(14)	N12-Cu2-N1	95.51(11)
N6^{i}-Ni1-N6	87.40(18)	N11-Cu2-N1	179.72(12)
N5^{i}-Ni1-N4^{i}	87.88(12)	N2-Cu2-S5^{i}	88.00(9)
N5-Ni1-N4^{i}	91.35(12)	N12-Cu2-S5^{i}	94.91(10)
N6^{i}-Ni1-N4^{i}	88.26(12)	N11-Cu2-S5^{i}	97.02(8)
N6-Ni1-N4^{i}	92.52(12)	N1-Cu2-S5^{i}	82.71(8)
N5^{i}-Ni1-N4	91.35(12)	N2-Cu2-S6^{ii}	85.86(8)
N5-Ni1-N4	87.88(12)	N12-Cu2-S6^{ii}	91.22(10)
N6^{i}-Ni1-N4	92.52(12)	N11-Cu2-S6^{ii}	85.25(8)
N6-Ni1-N4	88.26(12)	N1-Cu2-S6^{ii}	95.02(9)
N4#1-Ni1-N4	178.92(15)	S5i-Cu2-S6^{ii}	173.64(3)

^{i} -x, y, -z+1/2 ^{ii} -0.5+x, -0.5+y, z

Table S2. Intermolecular hydrogen bonds in the crystal network of $[\{Cu(pn)_2\}_2Ni(NCS)_6]_n \cdot 2nH_2O$ (**1**).

D-H	A	d(D-H) Å	D(H···A) Å	D(D···A) Å	∠DHA (°)
N1-H1B	N4 [–1/2+x,–1/2+y,z]	0.970	2.53	3.299(4)	136
N2-H2A	S4 [–x,y,1/2–z]	0.970	2.67	3.539(3)	149
N2-H2B	O7 [–1/2+x,–1/2+y,z]	0.970	2.30	3.190(4)	152
O7-H7A	S6 [x,1–y,–1/2+z]	0.82(2)	2.55(3)	3.291(3)	152(5)
O7-H7B	N5 [1/2–x,1/2+y,1/2–z]	0.82(3)	2.58(3)	3.314(4)	151(5)
N11-H11B	S5 [–x,–y,1–z]	0.970	2.75	3.550(3)	140
N12-H12A	O7 [–x,–1+y,1/2–z]	0.970	2.16	3.055(4)	153
N12-H12B	S4 [–1/2+x,–1/2+y,z]	0.970	2.79	3.639(3)	147

Table S3. Selected valence angles [°] for **2**.

N5-Ni1-N2	91.63(7)	N14-Cu2-N17	83.98(10)
N5-Ni1-$N2^{i}$	91.62(7)	N14-Cu2-N20	164.52(11)
N2-Ni1-$N2^{i}$	176.16(15)	N17-Cu2-N20	85.76(10)
N5-Ni1-N1	90.66(14)	N14-Cu2-N11	85.31(9)
N2-Ni1-N1	88.96(7)	N17-Cu2-N11	159.70(11)
$N2^{i}$ -Ni1-N1	88.96(7)	N20-Cu2-N11	100.77(10)
N5-Ni1-N4	175.42(14)	N14-Cu2-S4	99.43(9)
N2-Ni1-N4	88.46(7)	N17-Cu2-S4	99.11(8)
$N2^{i}$-Ni1-N4	88.46(7)	N20-Cu2-S4	93.61(8)
N1-Ni1-N4	93.92(14)	N11-Cu2-S4	99.63(7)
N5-Ni1-N3	89.37(15)	N6-Cu3-$S2^{ii}$	114.29(9)
N2-Ni1-N3	91.04(7)	N6-Cu3-$S2^{iii}$	114.29(9)
$N2^{i}$-Ni1-N3	91.04(7)	$S2^{ii}$-Cu3-$S2^{iii}$	94.54(4)
N1-Ni1-N3	179.97(14)	N6-Cu3-S3	120.37(14)
N4-Ni1-N3	86.05(14)	$S2^{ii}$-Cu3-S3	104.93(4)
		$S2^{ii}$-Cu3-S3	104.93(4)

^{i} x, -y+1/2, z ^{ii} -x+1, -y, -z+1 ^{iii} -x+1, y+1/2, -z+1

Table S4. Intermolecular hydrogen bonds in the crystal network of $[\{Cu^{II}(trien)\}_2Ni(NCS)_6Cu^{I}(NCS)]_n$ (**2**).

D-H	A	d(D-H) Å	D(H···A) Å	D(D···A) Å	∠DHA (°)
N17-H17A	S1 [-1/2+x, 1/2-y, 3/2-z]	0.98	2.58	3.561(3)	178
N20-H20A	S5 [3/2-x, -y, 1/2+z]	0.89	2.70	3.547(3)	159

Table S5. Selected valence angles [°] for **3**.

N5-Cu1-N27	93.62(14)	N4-Cu3-N14	177.33(12)
N5-Cu1-N24	176.98(13)	N4-Cu3-N11	94.60(14)
N27-Cu1-N24	83.91(12)	N14-Cu3-N11	84.01(11)
N5-Cu1-N21	96.60(13)	N4-Cu3-N17	94.84(13)
N27-Cu1-N21	130.61(19)	N14-Cu3-N17	84.11(11)
N24-Cu1-N21	83.76(11)	N11-Cu3-N17	124.68(12)
N5-Cu1-N30	98.85(13)	N4-Cu3-N20	98.18(13)
N27-Cu1-N30	114.16(17)	N14-Cu3-N20	84.49(11)
N24-Cu1-N30	83.77(11)	N11-Cu3-N20	117.18(14)
N21-Cu1-N30	111.71(17)	N17-Cu3-N20	115.10(12)
N2-Ni2-N2^{i}	180.0	N3^{i}-Ni2-N1^{i}	89.70(11)
N2-Ni2-N3^{i}	89.61(10)	N3-Ni2-N1^{i}	90.30(11)
N2^{i}-Ni2-N3^{i}	90.39(10)	N2-Ni2-N1	89.80(11)
N2-Ni2-N3	90.39(10)	N2^{i}-Ni2-N1	90.20(11)
N2^{i}-Ni2-N3	89.61(10)	N3^{i}-Ni2-N1	90.30(11)
N3^{i}-Ni2-N3	180.00(11)	N3-Ni2-N1	89.70(11)
N2-Ni2-N1^{i}	90.20(11)	N1^{i}-Ni2-N1	180.0
N2^{i}-Ni2-N1^{i}	89.80(11)		

^{i} -x+1,-y+1,-z

Table S6. Intermolecular hydrogen bonds in the crystal network of $[Cu(tren)(NCS)]_4[Ni(NCS)_6]$ (**3**).

D-H	A	d(D-H) Å	D(H···A) Å	D(D···A) Å	∠DHA (°)
N17-H17A	S1 [-x, 1-y, -z]	0.89	2.62	3.470(3)	159
N17-H17B	N5 [-x, 1-y, -z]	0.89	2.59	3.462(4)	166
N27-H27B	S2 [1-x, 1-y, -z]	0.89	2.60	3.390(4)	148

Table S7. Magnetic parameters for **1**.

g_{av}	J_{CuNi} [cm^{-1}]	zJ' [cm^{-1}]	TIP [cm^3 mol^{-1}]	CF[a] [cm^{-1}]	IMP[b] [%]	Method[c]	R[d]
{$NiCu_2$} unit							
2.201	0.059	0.019	461	0.757	1[e]	ms	0.0048
2.192	0.065	0.018	458	0.765	-	ms	0.0056
2.186	0.177	0.011	520 [e]	0.974	-	ms	0.0129
2.193	0.380	–0.046	455	-	-	s	0.0110
2.208	0.381	–0.045	462	-	1.6	s	0.0110
2.186	0.484	–0.055	520 [e]	-	-	s	0.0398
{Ni_2Cu_4} unit							
2.193	0.203	–0.011	909	0.597	-	s	0.0459
2.193	0.114	-	903	0.688	-	s	0.0468
2.193	0.344	–0.028	908	-	-	s	0.0444

[a] crystal field parameter, CF = D/3; [b] mononuclear impurities; [c] simulation method, ms – magnetization and susceptibility simultaneously, s – susceptibility only; [d] the “native” residual test functions of PHI software, $R_{ms} = \Sigma (M_{obs} - M_{calc})^2 \times \Sigma (\chi T_{obs} - \chi T_{calc})^2$, $R_s = (\chi T_{obs} - \chi T_{calc})^2$ [e] fixed parameter

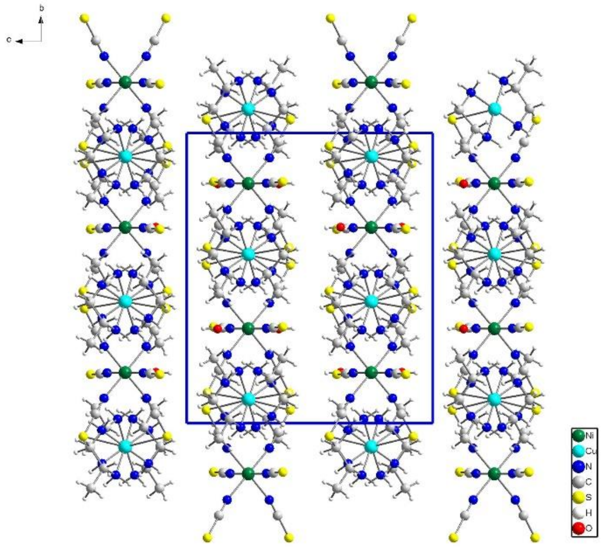

Figure S1. Packing of $[\{Cu(pn)_2\}_2Ni(NCS)_6]_n{\cdot}2nH_2O$ (**1**) along *a* axis shows robust *ab* layers. Analysis of formed interactions revealed that only the main set of atoms is involved into crystal network interactions and therefore for clarity of the figure minor population sets of atoms are omitted.

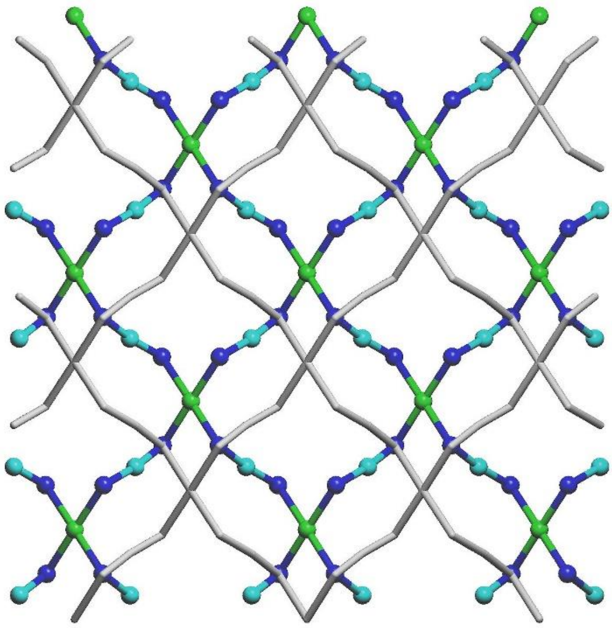

Figure S2. The arrangement of layers in **1** along *c* axis with the upper layer given in grey sticks and the lower layer is given in colored balls and sticks. This pattern clearly shows that they are shifted but the center of the upper layer is not positioned above the center of the network.

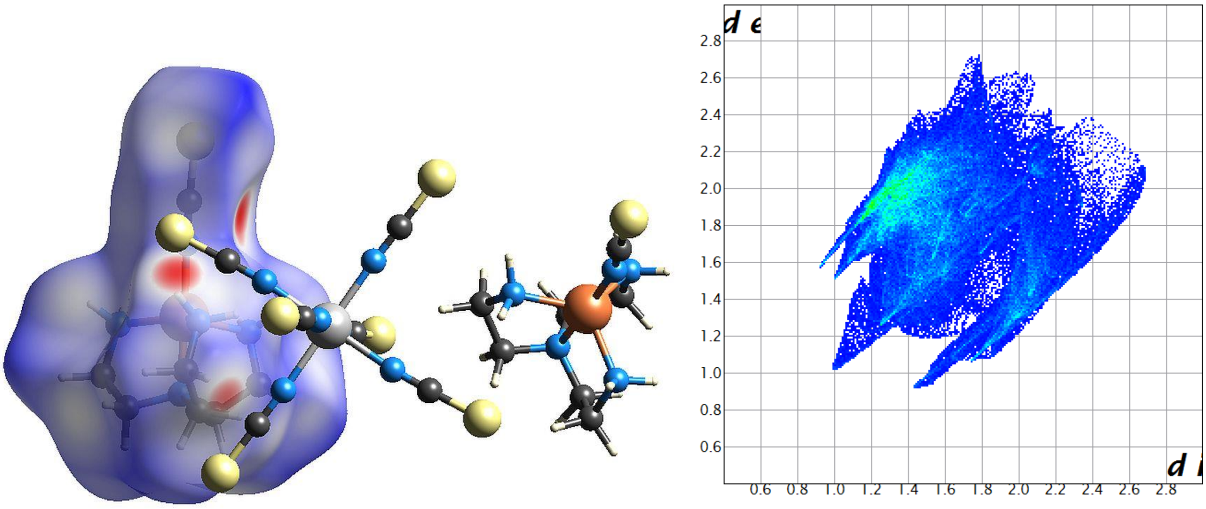
d e
2.8
2.6
2.4
2.2
2.0
1.8
1.6
1.4
1.2
1.0
0.8
0.6
d i
0.6 0.8 1.0 1.2 1.4 1.6 1.8 2.0 2.2 2.4 2.6 2.8

a.

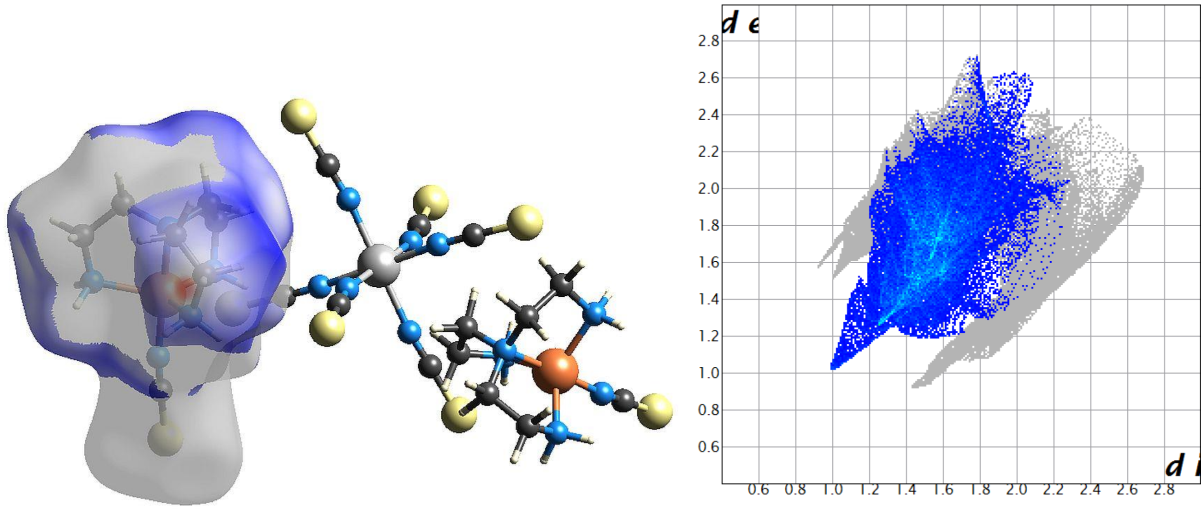
d e
2.8
2.6
2.4
2.2
2.0
1.8
1.6
1.4
1.2
1.0
0.8
0.6
d i
0.6 0.8 1.0 1.2 1.4 1.6 1.8 2.0 2.2 2.4 2.6 2.8

b.

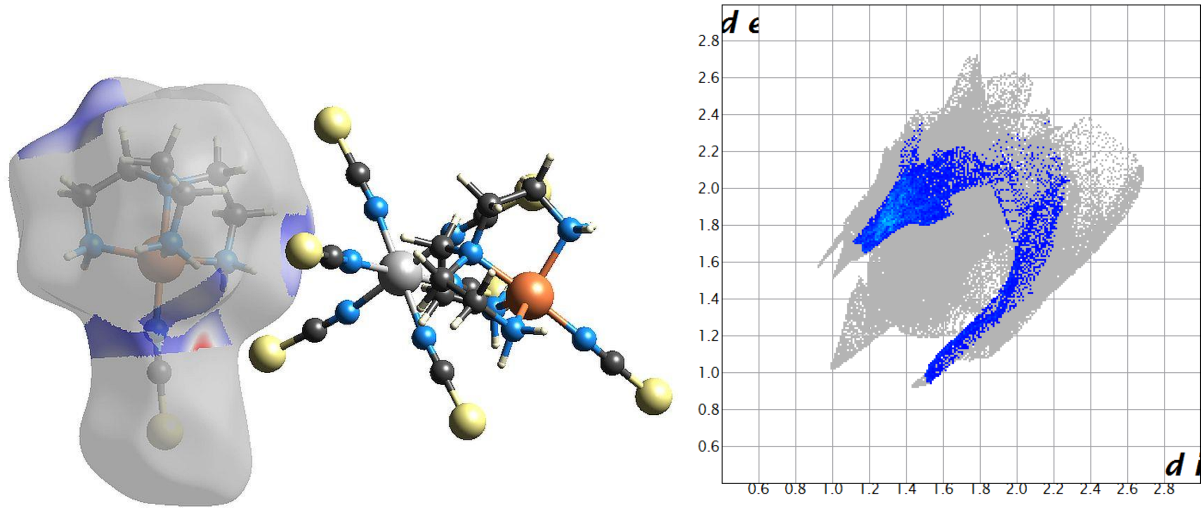
d e
2.8
2.6
2.4
2.2
2.0
1.8
1.6
1.4
1.2
1.0
0.8
0.6
d i
0.6 0.8 1.0 1.2 1.4 1.6 1.8 2.0 2.2 2.4 2.6 2.8

c.

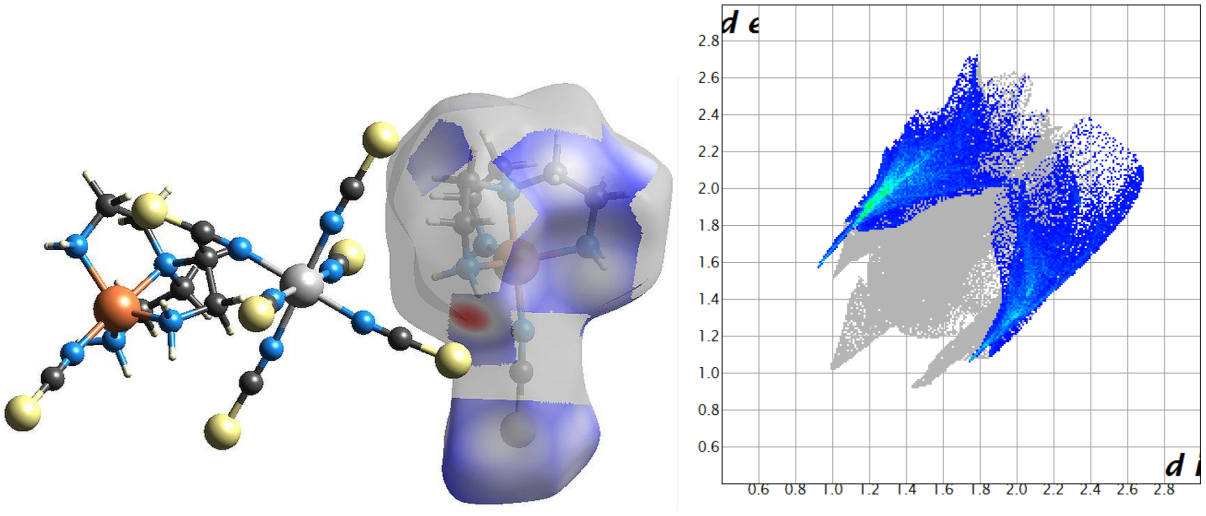
d e
2.8
2.6
2.4
2.2
2.0
1.8
1.6
1.4
1.2
1.0
0.8
0.6
0.6 0.8 1.0 1.2 1.4 1.6 1.8 2.0 2.2 2.4 2.6 2.8
d i

d.

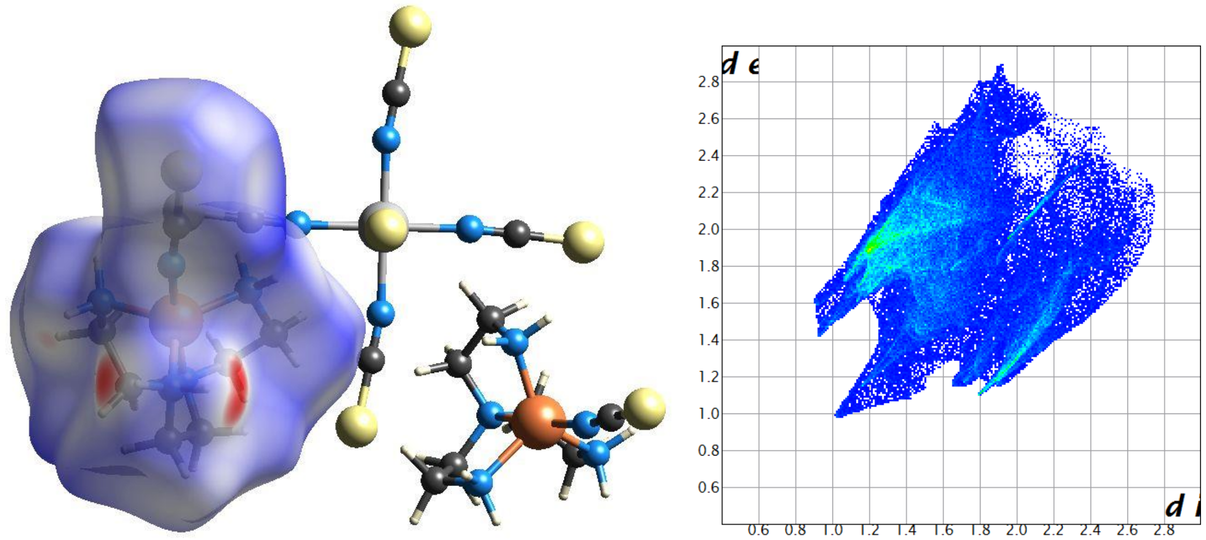
d e
2.8
2.6
2.4
2.2
2.0
1.8
1.6
1.4
1.2
1.0
0.8
0.6
0.6 0.8 1.0 1.2 1.4 1.6 1.8 2.0 2.2 2.4 2.6 2.8
d i

e.

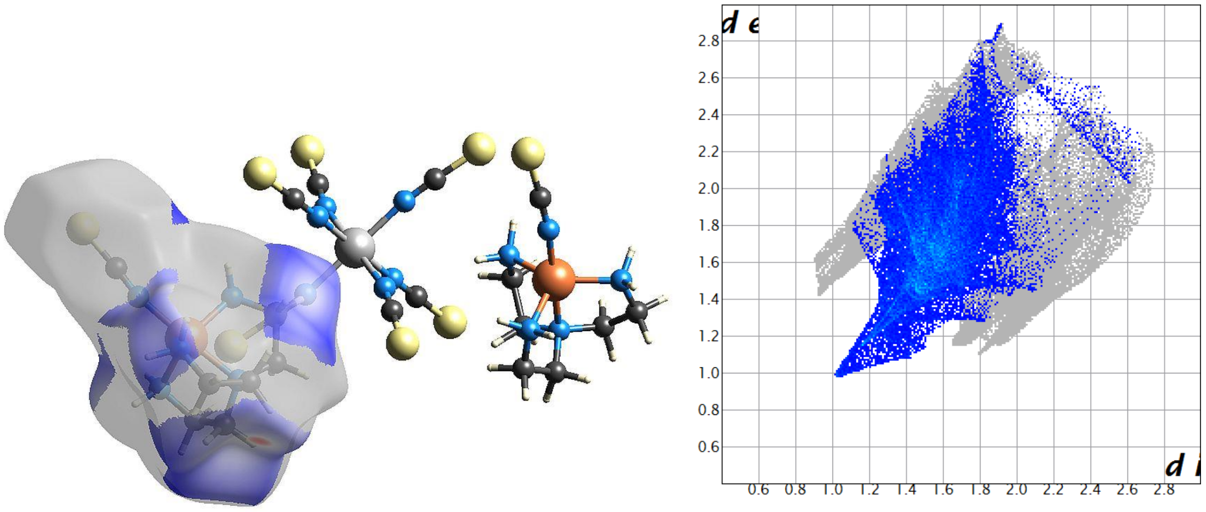
d e
2.8
2.6
2.4
2.2
2.0
1.8
1.6
1.4
1.2
1.0
0.8
0.6
0.6 0.8 1.0 1.2 1.4 1.6 1.8 2.0 2.2 2.4 2.6 2.8
d i

f.

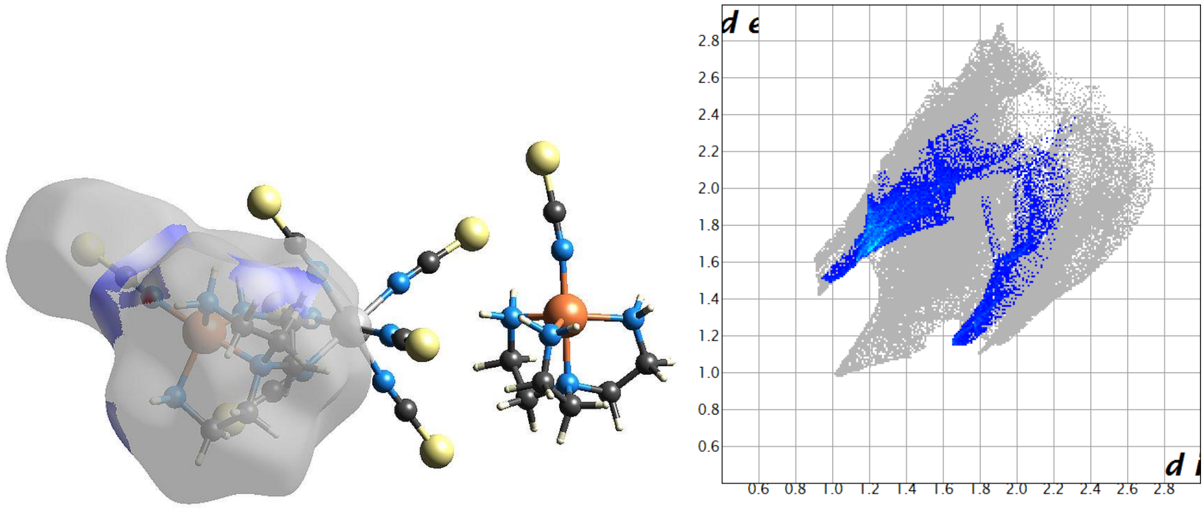

g.

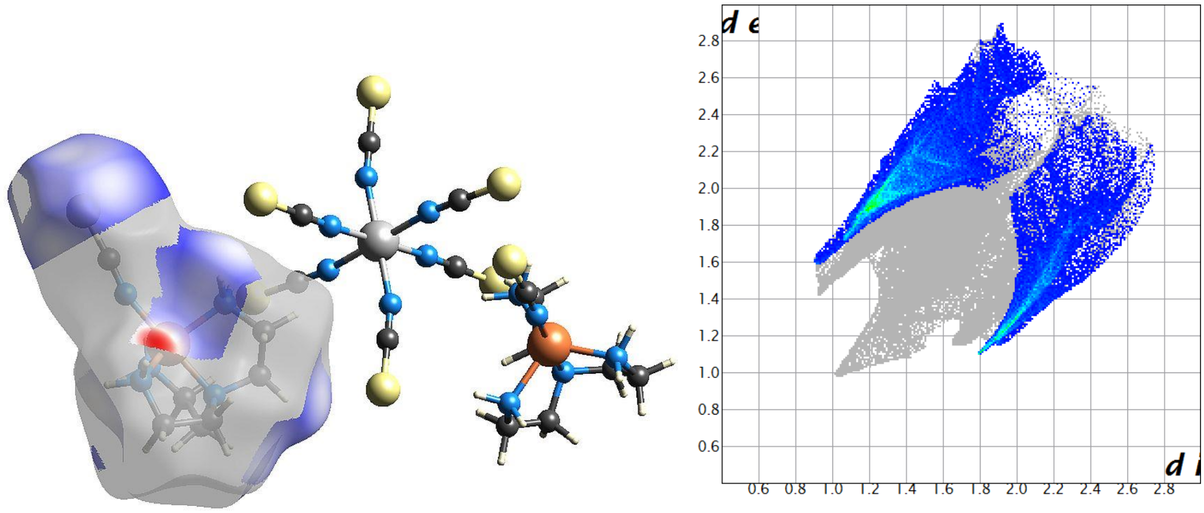

h.

Figure S3. Analysis of intermolecular interactions mapped onto Hirshfeld surface and given as a fingerprint in **3**: a. all atoms…all atoms for Cu1, b. H…H atoms for Cu1 (36.9%), c. N…H atoms for Cu1 (8.4%), d. S…H atoms for Cu1 (34.5%), e. all atoms…all atoms for Cu3, f. H…H atoms for Cu3 (31.4%), g. N…H atoms for Cu3 (9.9%), h. S…H atoms for Cu3 (38.6%).

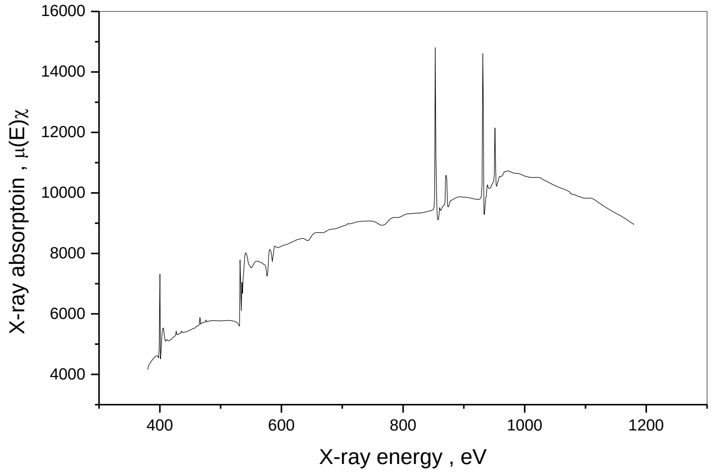

Figure S4. The XAS spectrum of $[\{Cu^{II}(trien)\}_2Ni(NCS)_6Cu^{I}(NCS)]_n$ (**2**).

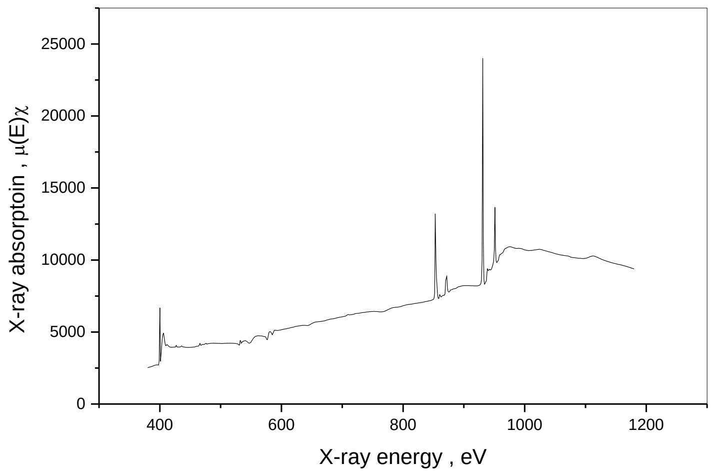

Figure S5. The XAS spectrum of $[Cu(tren)(NCS)]_4[Ni(NCS)_6]$ (**3**).

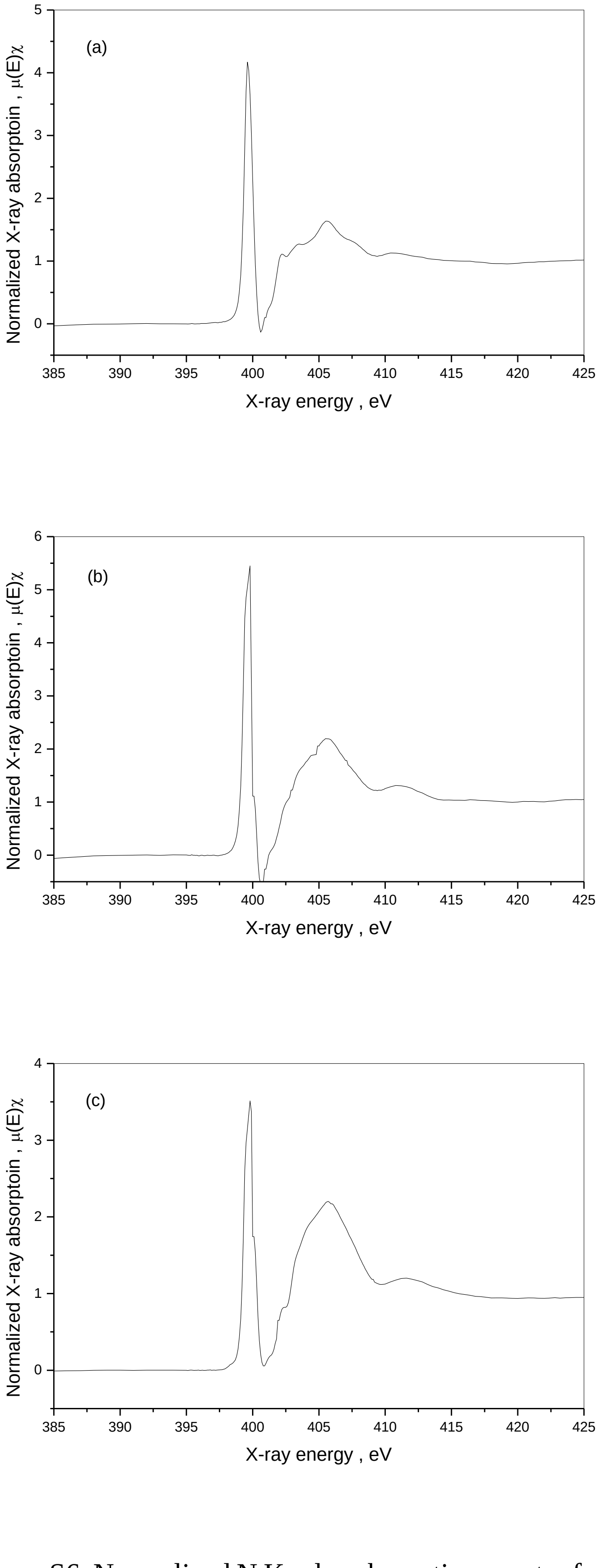

Figure S6. Normalized N K-edge absorption spectra for (**a**): $[\{Cu(pn)_2\}_2Ni(NCS)_6]_n\cdot 2nH_2O$ (**1**), (**b**): $[\{Cu^{II}(trien)\}_2Ni(NCS)_6Cu^{I}(NCS)]_n$ (**2**) and (**c**): $[Cu(tren)(NCS)]_4[Ni(NCS)_6]$ (**3**).
